# Distinct Injury Responsive Regulatory T Cells Identified by Multi-Dimensional Phenotyping

**DOI:** 10.3389/fimmu.2022.833100

**Published:** 2022-05-12

**Authors:** Fei Guo, Brandon Hancock, Alec Griffith, Hui Lin, Kaitlyn Howard, Joshua Keegan, Fan Zhang, Adam Chicoine, Laura Cahill, Julie Ng, James Lederer

**Affiliations:** ^1^ Department of Surgery, Brigham and Women’s Hospital and Harvard Medical School, Boston, MA, United States; ^2^ Ningbo Medical Centre Lihuili Hospital, Ningbo University, Ningbo, China; ^3^ Department of Pathophysiology, School of Basic Medical Sciences, Nanchang University, Nanchang, China; ^4^ Department of Critical Care Medicine, Qilu Hospital, Cheeloo College of Medicine, Shandong University, Jinan, China; ^5^ Human Immunology Center, Brigham and Women’s Hospital, Boston, MA, United States; ^6^ Department of Medicine, Division of Pulmonary and Critical Care Medicine, Brigham and Women’s Hospital, Boston, MA, United States

**Keywords:** Tregs, trauma immunology, CyTOF, T cell receptor diversity, single-cell RNA sequencing

## Abstract

CD4^+^ regulatory T cells (Tregs) activate and expand in response to different types of injuries, suggesting that they play a critical role in controlling the immune response to tissue and cell damage. This project used multi-dimensional profiling techniques to comprehensively characterize injury responsive Tregs in mice. We show that CD44^high^ Tregs expand in response to injury and were highly suppressive when compared to CD44^low^ Tregs. T cell receptor (TCR) repertoire analysis revealed that the CD44^high^ Treg population undergo TCRαβ clonal expansion as well as increased TCR CDR3 diversity. Bulk RNA sequencing and single-cell RNA sequencing with paired TCR clonotype analysis identified unique differences between CD44^high^ and CD44^low^ Tregs and specific upregulation of genes in Tregs with expanded TCR clonotypes. Gene ontology analysis for molecular function of RNA sequencing data identified chemokine receptors and cell division as the most enriched functional terms in CD44^high^ Tregs versus CD44^low^ Tregs. Mass cytometry (CyTOF) analysis of Tregs from injured and uninjured mice verified protein expression of these genes on CD44^high^ Tregs, with injury-induced increases in Helios, Galectin-3 and PYCARD expression. Taken together, these data indicate that injury triggers the expansion of a highly suppressive CD44^high^ Treg population that is transcriptionally and phenotypically distinct from CD44^low^ Tregs suggesting that they actively participate in controlling immune responses to injury and tissue damage.

## Introduction

Traumatic injury causes sudden, non-infectious tissue damage that initiates a complex immune response aimed at controlling excessive inflammation and maintaining immunological tolerance against exposure to sequestered self-antigens (1). When unchecked, the pro-inflammatory response to trauma, manifesting clinically as the systemic inflammatory response syndrome (SIRS), can lead to significant morbidity and mortality from shock and organ failure (2, 3). Key immune features of SIRS include augmented Toll-like receptor (TLR) responsiveness, enhanced granulopoiesis, and inflammasome activation in innate immune cell types (4, 5). In contrast, the adaptive immune response to injury is skewed towards the compensatory anti-inflammatory response syndrome (CARS) phenotype that is characterized as enhanced regulatory T cell (Treg) activity, increased Th2-type cytokine production by T cells, and reduced antigen-specific Th1 responses (6–11). Imbalances in these innate and adaptive immune response phenotypes are central to the loss of immune homeostasis that can predispose people to secondary infections or inflammation-mediated complications following severe injury (12). Thus, there is a critical need to understand the complex immune regulatory responses that occur in response to traumatic injuries to forward the development of beneficial immunotherapies.

Tregs are vital to the maintenance of peripheral immune tolerance (13–15). In mice, Tregs were identified as being acutely activated by injury, and are the primary adaptive immune cell subset that controls the pro-inflammatory SIRS phenotype (12, 16). Furthermore, injury enhances the immune suppressive potency of Tregs from injury-site draining lymph nodes, but not Tregs from the spleens in a mouse burn injury model (10). This compartmentalized response by Tregs, as well as their rapid activation by injury, supports the possibility that Tregs can react to danger associated molecular patterns (DAMPs), inflammatory cytokines, stress, or protein antigens that are exposed by tissue damage.

Our group has recently demonstrated that CD44^high^ Tregs are highly reactive to burn trauma in mice, and show expansion and upregulation of Treg effector molecules such as CTLA4, ICOS, and GITR (17). Moreover, this CD44^high^ Treg subpopulation is similar to those found on expanded memory Tregs (mTregs) in inflammatory and autoimmune diseases like multiple sclerosis (18), coronary artery disease (19), nephritis (20), type I diabetes (21), allergy (22) and obesity (22). Little is known about how mTregs are activated or their biological functions, although it is thought that mTregs may react to self-antigens and function to control excessive inflammation at sites of tissue damage (23). Given that trauma induces rapid activation of mTregs, we hypothesize that a pool of mTregs is specifically reacting to DAMPs or other antigens that are released or exposed to immune cells following tissue trauma. To address this hypothesis, unsupervised transcriptomic and single-cell technologies were used to comprehensively characterize injury-reactive Tregs with the objective, to contribute novel insights into Treg biology and their phenotypic response to cell and tissue damage.

## Materials and Methods

### Study Design

This project used unbiased systems immunology approaches including RNA sequencing, mass cytometry (CyTOF), and TCR repertoire analytical methods to test the hypothesis that the Tregs that respond to injury represent a subset of CD4+FoxP3+ T cells in mice. All experiments were performed using a well-defined mouse burn trauma model following ARRIVE guidelines. Age- and sex-matched mice were randomized into control and experimental groups, with sample sizes chosen based on statistical power analysis and previous experience. Experimental replication for each experiment is indicated in the figure legends. The investigators were not blinded, and no data were excluded from analysis.

### Mice

C57BL/6 wild-type (stock #000664), Foxp3^DTR^ (stock #016958) and BALB/c (stock #000651) mice were purchased from Jackson Laboratory (Bar Harbor, ME). Eight to16-weeks old mice were acclimated for at least 1 week before being used for experiments. All procedures performed in this study were reviewed and approved by the Brigham and Women’s Hospital IACUC (2020N000458) and were found to be in accordance with guidelines set by the U.S. Department of Agriculture (Washington, DC) and the National Institutes of Health (Bethesda, MD).

### Mouse Injury Model

A mouse burn injury model was used to model traumatic injury in mice as previously described (24). Briefly, mice were anesthetized by intraperitoneal (IP) injection with 125 mg/kg ketamine (Fort Dodge Animal Health, Fort Dodge, IA) and 10 mg/kg xylazine (Lloyd Laboratories, Shenandoah, IA). Buprenorphine at 0.6mg/kg was injected subcutaneously at the time of injury. The mice had their dorsal fur shaved and were placed in a plastic mold exposing 20% of their total body surface area. Injury was induced by immersing the exposed part of the dorsum for 9 seconds in a 90˚ C water bath. This approach causes a full-thickness and well-demarcated anesthetic injury due to complete loss innervation. Uninjured mice underwent the same procedure but were exposed to room temperature water for 9 seconds. All animals were resuscitated with an IP injection of 1 mL of 0.9% pyrogen-free normal saline containing ANTISEDAN (atipamezole, Zoetis, US) at 1mg/kg. The mortality from this burn trauma model is less than 2%.

### Lymph Node Cell Preparations

Mice were euthanized by CO_2_ asphyxiation. Injury-site draining lymph nodes (axillary, brachial, and inguinal) were harvested and immediately placed in ice-cold culture medium (RPMI 1640 supplemented with 5% heat-inactivated fetal bovine serum (FBS), 1 mM glutamine, 10 mM HEPES, 100 μM nonessential amino acids, penicillin/streptomycin/fungizone, and 50 μM 2-mercaptoethanol, all purchased from Gibco-Invitrogen, Grand Island, NY). To prepare single cell suspensions, the lymph nodes were mechanically minced through sterile 70- μm cell strainers. Cell preparations were washed twice in culture medium and then strained to remove debris.

### Flow Cytometry and Cell Sorting

Fluorescent conjugated antibodies were purchased from BioLegend (San Diego, CA) or eBioscience (Waltham, MA): APC/Cy7-labeled anti-CD3 (145-2C11), APC-labeled anti-CD44 (IM7), eFluor450-labeled anti-FoxP3 (FJK-16s), and PE/Cy7-labeled anti-CD4 (GK1.5), FITC-labeled anti-CD25(PC61). FITC-labeled Anti-Mouse TCR Vβ Screening Panel was purchased from BD bioscience. Zombie NIR™ Fixable Viability Kit (BioLegend) was used for cellular viability staining. Flow cytometry stains were performed in PBS with 1% BSA and 0.1% sodium azide at room temperature. Cells were plated and Fc-block reagent (TruStain FcX Fc receptor blocking reagent, BioLegend, San Diego, CA) was added for 10 min to minimize nonspecific antibody staining before adding cell-surface fluorochrome-labeled antibodies. For intracellular stains, cells were permeabilized using the FoxP3/transcription factor staining buffer set from eBioscience (eBioscience/ThermoFisher Scientific, Waltham, MA). Stained samples were fixed in 4% PFA in PBS, washed by centrifugation, then reconstituted in PBS for flow cytometry analysis on a MACS-Quant Analyzer (Miltenyi Biotech, San Diego, CA). Data analysis was performed using the FlowJo software program (Tree Star, Ashland, OR). The flow cytometry gating schemes for T cells, CD4^+^ and CD4^-^ T cells, CD44^high^ and CD44^low^ Tregs as well as TCRVβ are presented in **Supplemental Figures 6**, **7**. For sorting Tregs, the stains were performed in sorting buffer (RPMI 1640 without phenol red supplemented with 0.5% BSA, 1 mM glutamine, 10 mM HEPES, 100 μM nonessential amino acids, penicillin/streptomycin/fungizone, and 50 μM 2-mercaptoethanol). CD4+ T cells were purified by negative selection using Miltenyi MACS CD4^+^GFP^+^ Tregs or CD4^+^GFP^+^CD44^high^ and CD4^+^GFP^+^CD44^low^ cells were sorted respectively according to experimental requirements.

### Treg Suppression Assay

CD4^+^CD25^-^ T cells (Tconv) for Treg functional assays were negatively selected using LS Columns (Miltenyi Biotec, 130-042-401) with MidiMACS™ Separator (Miltenyi Biotec,130-042-302) by staining the cells with Biotin-labeled anti-mouse CD8a (BioLegend 100704), CD25 (BioLegend 102004), TER119 (BioLegend 116204), CD45R/B220 (BioLegend 103204), CD49b (BioLegend 108904), and CD11b (BioLegend 101204). The negatively selected cells were collected and stained with Zombie NIR™ Fixable Viability kit and then CellTrace™ Violet Cell Proliferation Kit (ThermoFisher, C34571) for the Treg functional assay. CD44^high^ and CD44^low^ Tregs were sorted respectively from sham or day 7 after injury Foxp3^DTR^ mice, co-cultured with CellTrace Violet labeled Tconv, and stimulated with mouse T cell activator anti-CD3/CD28 beads (Gibco, 11452D). Purified Tconv (8 x 10^4^) were co-cultured with CD44^high^ or CD44^low^ Tregs at 1:1, 1:2, 1:4, 1:8 Treg : Tconv ratios or control Tconv alone. Proliferation of CD3/CD28 bead stimulated Tconv without added Tregs was set as control proliferation with 0% suppression. Proliferation was measured using the CellTrace Violet dilution assay by flow cytometry after 4 days co-culture that detects proliferated cells by reduced fluorescence intensity in cells that undergo one or more cell divisions. Suppression% was calculated as: $100-\left(\frac{\text{Percent proliferated cells in Treg}:\text{Tconv}}{\text{Percent proliferated cells Tconv only}}\times 100\right)$

### Depletion of Tregs in FoxP3^DTR^ Mice, Adoptive Transfer of Tregs, and Secondary *Pseudomonas aeruginosa* Lung Infection Model

Foxp3^DTR^ mice were treated with 40ng/kg diphtheria toxin (DT) at 2h and 24h after burn injury to deplete Tregs. Male C57BL/6 mice underwent burn trauma injury. At 7 days after injury, CD4 T cells were purified from injury-site draining lymph nodes (axillary, brachial, and inguinal) into CD44^high^ or CD44^low^ Tregs using anti-CD25 and anti-CD44 antibodies. Tregs from injured mice were transfused into the anesthetized injured Foxp3^DTR^ mice depleted of Tregs at 50,000 cells/mouse by intracardiac injection in 0.2 ml of PBS. Mice were subsequently challenged with intranasal *Pseudomonas aeruginosa* 1 day after Treg-transfusion. Survival was monitored over a week period. For lung infections, *Pseudomonas aeruginosa* (ATCC 27853) were grown for 16 hours with gentle agitation in brain-heart infusion (BHI) broth medium at 37°C and were harvested by centrifugation at 450 x *g* for 10 minutes, then washed once with PBS by centrifugation. Based on absorption spectroscopy measurements (ABS_600_), bacteria were diluted to contain 3-5 x 10^7^ CFUs/ml in PBS. Mice were anesthetized by IP injection using Ketamine/Xylazine at 125/10 mg/kg. Mice were then held with their nares upright, and 40 μl of bacteria suspension was administered by intranasal route (1.2-2 x 10^6^ CFUs). This inoculum dose was found to cause 50% mortality in male C57BL/6 mice over a 7 day period, with deaths first occurring at days 2-3 after infection. Bacteria CFUs were quantified by drop-plating of serial dilutions on Luria broth (LB) agar plates, and colonies were counted the following day after incubating the plates at 37°C.

### RNA Sequencing of Sorted FoxP3^+^ T Cell Populations

Lymph node CD4^+^CD44^high^FoxP3-GFP^+^ and CD4^+^CD44^low^FoxP3-GFP^+^ T cells or FoxP3-GFP^+^ T cells were purified by FACS sorting at 7 days after injury from injured or uninjured Foxp3^DTR^ mice (strategy shown in **Supplementary Figure S7**). RNA was purified from 50,000 cells from each sample using the RNeasy Micro Kit (Qiagen, CA) according to manufacturer’s protocol. RNA sequencing (RNAseq) was performed by the Molecular Biology Core Facilities (MBCF) at Dana-Farber Cancer Institute (DFCI). cDNA was synthesized using Takara SMART-Seq v4 PLUS kit and was fragmented to ~150bp by Covaris Adaptive Focused Acoustics^®^-AFA^®^ technology. The cDNA library prepared with Swift 2S™ Turbo DNA library kit from 2ng of cDNA was submitted for next-generation sequencing (NGS).

### RNAseq Data Processing and Analysis

Analysis of RNA sequencing data was performed using Visualization Pipeline for RNA-seq (VIPER) workflow (25). The VIPER workflow provides sample to sample correlation and sample to feature heatmap plots to judge the correlation and clustering patterns of all samples (**Figures S2A, B**). Gene count stabilized variance of RNAseq data was determined using the *vst* function of DESeq2 (26). Principal component analysis (PCA) plots were generated based on the variance stabilized counts. The top 2000 genes that were most differentially expressed in CD44^high^ versus CD44^low^ Tregs were identified using DESeq2 and input into the gene ontology (GO) molecular function term enrichment analysis webtool (27–29). Heat maps were generated using gene count Z-scores of highly variable cytokines, cell surface markers, and transcription factor genes. Hierarchical clustering of samples was done using the *hclust* function in R. Differentially expressed genes between Treg subsets and the effects of injury on gene expression were identified by DESeq2 and volcano plots showing differential expression of genes were generated using the Enhanced Volcano R package (30).

### RNA-Based TCR Repertoire Sequencing

Viable CD4^+^CD44^high^GFP^+^ and CD4^+^CD44^low^GFP^+^ cells were sorted from lymph nodes harvested at 7-days after injury from burned or uninjured Foxp3^DTR^ mice (cell numbers are detailed in **Supplementary Table 1**). All RNA extraction, cDNA synthesis, amplification, NGS library preparation and sequencing were performed by iRepertoire, Inc. (Huntsville, USA), RNA was extracted from FACS sorted cells using Qiagen’s RNeasy Micro kit with DNase Treatment. One-third of the total RNA from each sample were used for the construction of mouse TCRα and β chain libraries by reverse transcription polymerase chain reaction (RT PCR) using Qiagen’s OneStep RT PCR mix (Qiagen). First strand cDNA was selected, and unused primer was removed by SPRIselect beads (Beckman Coulter) followed by a second round of binding and extension with the V-gene primer mix. After binding and extension, SPRIselect beads were used to purify the first and second strand synthesis products. Library amplification was performed with a pair of primers that are specific for communal sites engineered onto the 5’ end of the C- and V- primers used in first and second strand synthesis. The final constructed library includes Illumina dual index sequencing adapters, a 10-nucleotide unique molecular identifier region, and an 8-nucleotide internal barcode associated with the C-gene primer. The amplified libraries were multiplexed and pooled for sequencing on the Illumina MiSeq platform using a 600-cycle kit and sequenced as 250 pair-end read. The portion of TCRA and TCRB receptor genes that were sequenced included the second framework region stretching to the beginning of the TCR constant region, including the CDR2 and CDR3 regions.

### TCR Sequence Analysis

Sequencing of raw data were analyzed using the previously described iRmap program (11, 31). Sequence reads were de-multiplexed according to both Illumina dual indices incorporated during the amplification process and barcode sequences at the 5’ end of reads from the constant region. Reads were then trimmed according to their base qualities with a 2-basesliding window. If either quality value in this window was lower than 20, this sequence stretching from the window to the 3’ end, was trimmed out from the original read. Trimmed pair-end reads were joined together through overlapping alignment with a modified Needleman-Wunsch algorithm (32). If paired forward and reverse reads in the overlapping region were not perfectly matched, both forward and reverse reads were thrown out without further consideration. The merged reads were mapped using a Smith-Waterman algorithm to germline V, D, J and C reference sequences using an IMGT reference library. To define the CDR3 region, the position of CDR3 boundaries of reference sequences from the IMGT database was migrated onto reads through mapping results, and the resulting CDR3 regions were extracted and translated into amino acids.

### TCR Repertoire Data Analysis

Treemap plots were generated by iRweb tools (iRepertoire, Inc. Huntsville, USA). V, D and J gene usage and CDR3 sequences were identified and assigned. In treemap plots, each unique CDR3 is shown as a colored rectangle. The size of each rectangle corresponds to the abundance of each CDR3 within the repertoire and the positioning is determined by the V region usage. To compare the relative diversity of TCR libraries, we used iRweb tools to generate diversity-50 (D50) values, diversity plots, and diversity index (Di) values. D50 is the percent of unique TCR CDR3 sequences that account for the cumulative 50% of the total CDR3s counted in the sample. $D50=\;\frac{number\;of\;uCDR3\;sequences\;in\;top\;50\%\;\times \;100\;}{number\;of\;uCDR3\;in\;top\;10,000\;CDR3\;sequences}$ . Low D50 values indicate higher clonality. The diversity index (Di) was calculated as: $Di=\left[1-\;\frac{\sum^{}n(n-1)}{N(N-1)}\;\right]\times 100$ . TCR diversity index values are proportional to the values where high values indicate high diversity, while low values indicate low diversity. Diversity index plots were generated by plotting the percentage of total TCR reads versus the percentage of unique CDR3 sequences. The curve illustrates the overall diversity of the population with full diversity represented by the dashed line in the middle. The higher the TCR diversity in the cell population, the closer the curve will be to the dashed line in the middle. The D50 values are indicated on these diversity index plots to illustrate the differences in TCRA and TCRB clonality in Treg populations from uninjured and injured mice.

### Preparation of scRNAseq and scTCRseq Libraries

For single cell RNAseq (scRNAseq) and single cell TCRseq (scTCRseq), viable lymph node FoxP3 GFP^+^ cells were sorted from injured or uninjured Foxp3^DTR^ mice at 7 days after injury. The sorted FoxP3 GFP^+^ cells were washed and resuspended in sorting buffer at a cell concentration of 1000 cells/µL. About 17,000 mouse cells were loaded onto a 10x Genomics Chromium™ instrument (10x Genomics) according to the manufacturer’s recommendations. The scRNAseq libraries were processed using Chromium™ single cell 5’ library & gel bead kit (10x Genomics). Matched scTCRseq libraries were prepared using 2 µL of post cDNA amplification material and Chromium Single Cell V(D)J Enrichment Kit, Mouse T Cell. Quality controls for amplified cDNA libraries and final sequencing libraries were performed using Bioanalyzer High Sensitivity DNA Kit (Agilent). The sequencing libraries for scRNAseq and scTCRseq were normalized to 4nM concentration and pooled using a volume ratio of 4:1. The pooled sequencing libraries were sequenced on Illumina NovaSeq S4 300 cycle platform. The sequencing parameters were: Read 1 of 150bp, Read 2 of 150bp and Index 1 of 8bp. The sequencing data were demultiplexed and aligned to GRCm38 using cell ranger version 3.1.0 pipeline (10x Genomics).

### Single-Cell RNAseq Data Analysis

scRNAseq reads were aligned to the mouse reference genome (GRCm38) with the Ensembl GRCM28.91 GTF file using Cell Ranger (10x Genomics; sample statistics in **Supplementary Table 3**). The uninjured and 7D after injury samples were aggregated using the *aggr* function in Cell Ranger. Cell Ranger gene expression matrices were further analyzed with Seurat (v3.2.2). Matrices were filtered to exclude low-quality cells; cells with < 500 or > 4000 unique features. Cells with > 7% reads mapping to the mitochondrial genome were filtered out (thresholds chosen using analysis shown in **Supplementary Figures S4 C, D**). Feature counts were normalized using the *NormalizeData* function of Seurat. Per standard methods, we identified the 2,000 most variable genes and did PCA on these genes. Gene counts were scaled using the *ScaleData* function of Seurat. Clustering was done using the *FindNeighbors* (dims = 1:20) and *FindClusters* (resolution = 0.5) functions in Seurat. Cluster 6 was then removed as these cells expressed B cells markers. TCR clonotype metadata from the output of Cell Ranger VDJ was added to each cell using custom scripts. Cells with a CDR3 clonotype found in 2 or more cells were labeled as “Expanded”. Clustering was redone with parameters to generate many clusters, *FindNeighbors* (dims = 1:25) and *FindClusters* (resolution = 2.0) functions in Seurat. Clusters composed of 15% or more expanded cells were labeled as “Expanded” phenotype. Normalized expression was visualized with t-SNE plot projections and violin plots using Seurat.

### Data Processing of scTCRseq Libraries

For scRNAseq, the raw sequencing data generated by Cell Ranger 3.1.0 were loaded into the third-party tools for secondary analysis: Loupe Cell Browser 4.1.0 and Loupe VDJ Browser 3.0.0 (https://www.10xgenomics.com/). TCR sequencing data were aligned to the mm10 reference genome and RefSeq gene models using Cell Ranger VDJ TCA and TCB sequences from individual cell were used to infer clonotypes. The clonotype comparison feature in Loupe Cell Browser was then applied to pool TCR clonotypes across groups by matching CDR3 amino acids of both TCRA and TCRB. TCR clonotypes were designated as “expanded” if the paired CDR3 sequences were found in 2 or more cells.

### Mass Cytometry (CyTOF)

CyTOF staining was performed at room temperature as previously described (33). Cells harvested from lymph nodes were incubated with cisplatin live/dead stain for 2 min (Fluidigm) and blocked with TruStain FcX Fc receptor blocking reagent for 10 min before antibody staining. For surface staining, cells were stained with the CyTOF antibody cocktail for 30 min. After washing twice with staining buffer (calcium/magnesium-free PBS, 0.2% BSA, 0.05% sodium azide), cells were fixed and permeabilized using eBioscience™ Fixation/Permeabilization kit (Invitrogen). Subsequently, cells were barcoded using a palladium-based barcode reagent (33). After washing out excess barcode reagent, samples were pooled together and stained with intracellular CyTOF antibodies. Details of all antibodies used for CyTOF staining are listed in **Supplementary Table 4**. Single-cell CyTOF data was collected using a Helios mass cytometer (Fluidigm). Data normalization and deconvolution of barcoded staining data was conducted using the normalizer and the single-cell-debarcoder software developed in the Nolan Lab (Stanford) (33, 34). Data analysis was conducted using Cytobank (35). A nonlinear dimensionality reduction algorithm, viSNE, was run on all markers for dimensionality reduction (36). A hierarchical clustering algorithm, SPADE was run on viSNE parameters to cluster cells for phenotypic identification (37).

### Statistical Analysis

Statistical analysis was performed using GraphPad Prism 8.4.3 (GraphPad Software Inc. San Diego, CA, USA) or custom R scripts for RNAseq data. Data comparing multiple groups were analyzed by one-way ANOVA with Dunnett statistical hypothesis testing to correct for multiple comparisons and assuming Gaussian distribution of residuals. Data consisting of one variable and two factors were analyzed by two-way ANOVA and the Sidak or Tukey tests were applied for multiple comparison correction as appropriate. For protein expression validation studies by CyTOF, two-tailed unpaired t-tests were applied, and Welch’s correction was used if two populations had different standard deviations. Analyzed data with P <0.05 were considered as significantly different.

## Results

### Trauma Induces Expansion of Highly Suppressive CD44^high^ Tregs

**Figure 1:**
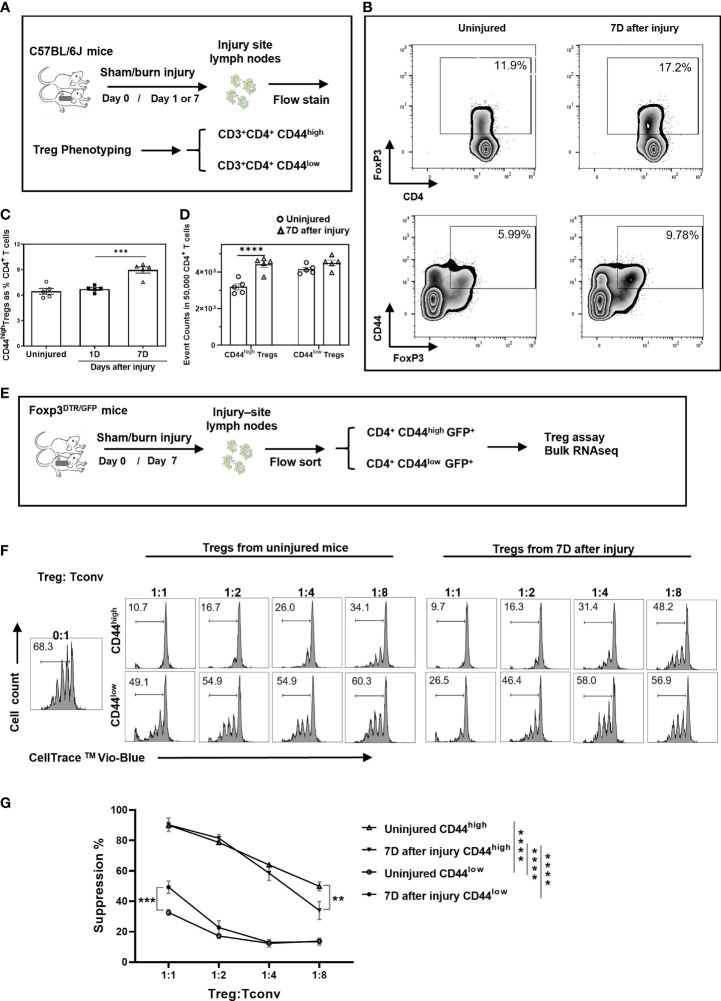
Burn trauma induces the expansion of CD44^high^ Tregs in injury-site draining lymph nodes that more potently suppress T cell proliferation than CD44^low^ Tregs. **(A)** Experimental scheme for mouse Treg phenotyping by flow cytometry; **(B)** Representative flow cytometry contour plots illustrating Tregs and CD44^high^ Tregs in CD4^+^ T cells in injured and uninjured mice. **(C)** CD44^high^ Tregs are significantly increased in injury-site draining lymph nodes at 7 days (Δ), but not at 1 day (■) after injury compared to uninjured controls (○). Data are presented as mean ± standard error of the mean (SEM) and analyzed by one-way analysis of variance (ANOVA), P=0.0001. Significant comparisons by Dunnett’s multiple comparisons test are denoted by *** (uninjured versus 7 days after burn), P=0.0002. **(D)** CD44^high^ and CD44^low^ Treg cell events in equal-sampled CD4^+^ T cells. Flow cytometry data represent two independent experiments (n=5 per group). Data were analyzed by two-way ANOVA, interaction P=0.0117. Significant comparisons by Sidak’s multiple comparisons test are denoted by **** (uninjured control versus 7 days after injury in CD44^high^ Tregs), P<0.0001. **(E)** Experimental scheme for Treg functional assay and bulk RNA sequencing. **(F)** CD4^+^CD25^-^ T cells (Tconv) FACS sorted from lymph nodes were labeled with CellTrace Violet and co-cultured with FACS sorted CD44^high^ and CD44^low^ Tregs from uninjured or injured Foxp3^DTR^ mice at the indicated cell concentration ratios. CellTrace Violet dilution was used to calculate percentage of proliferating Tconv cells after 4 days of co-culture with anti-CD3/CD28 antibody-coated T cell activation beads. Representative Treg suppression plots are shown. **(G)** Plots summarizing Treg suppressive activity. Error bars represent the mean ± SEM of n = 3 replicate wells. Data were analyzed by two-way ANOVA, interaction P<0.0001. Significant comparisons by Dunnett’s multiple comparisons test are indicated by **P<0.01, ***P<0.001 and ****P<0.0001.

We first assessed the function of CD44^high^ Tregs in a mouse model of burn injury. Injury-site draining lymph nodes were harvested from mice to measure CD44^high^ and CD44^low^ CD4^+^FoxP3^+^ T cell (Treg) abundances at 1 and 7 days after burn trauma injury using the scheme shown in **Figure 1A**. By day 7, there was a significant increase in CD44^high^ Tregs in injury-site draining lymph nodes compared to uninjured controls (**Figures 1B, C**). In contrast, there was no difference in the number of CD44^low^ Tregs between injured and control mice (**Figure 1D**). Next, we utilized a fluorescence-activated cell sorting (FACS) approach to purify CD44^high^ and CD44^low^ Treg subsets to assess Treg function and for subsequent RNA sequencing studies (**Figure 1E**). In Treg-mediated suppression assays, both CD44^high^ and CD44^low^ Tregs significantly suppressed the proliferation of CD4^+^CD25^-^ T cells (Tconv). The CD44^high^ Tregs were identified as being much more potent than CD44^low^ Tregs at suppressing T cell proliferation and injury did not further enhance the suppressive function of CD44^high^ Tregs (**Figures 1F, G**). However, we did observe that CD44^low^ Tregs from injured mice were significantly more suppressive at a 1:1 Treg to Tconv cell ratio. Taken together, both Treg subsets are affected by injury showing either expansion (CD44^high^) or enhanced Treg suppressive function (CD44^low^).

Because infectious complications are a leading cause of death in trauma patients (38, 39), we next tested whether CD44^high^ or CD44^low^ Tregs were beneficial or harmful in a secondary infection after burn injury (**Figure S1A**). In brief, Foxp3^DTR^ mice were Treg depleted by administration of diphtheriae toxin after burn trauma as previously described (40). Treg depletion was validated by flow cytometry (**Figure S1B**). CD44^high^ and CD44^low^ Tregs sorted from injury-site draining lymph nodes of burn trauma mice were then adoptively transferred into Treg-depleted mice 2 days after injury. Mice were challenged intranasally with *Pseudomonas aeruginosa* (1.2-2 x 10^6^ CFU per mouse) the next day and were observed over a week for survival (9, 10, 12). Injured mice given CD44^high^ Tregs demonstrated a trend towards lower survival (43%) compared to mice given CD44^low^ Tregs (75%, probability of survival, P=0.0617) (**Figure S1C**). Thus, CD44^high^ Tregs suppress anti-microbial immune function more so than CD44^low^ Tregs and their expansion may increase susceptibility to secondary infections following traumatic injury.

### RNA Sequencing Analysis of CD44^high^ and CD44^low^ Treg Subsets Reveals Distinct Molecular Signatures

**Figure 2:**
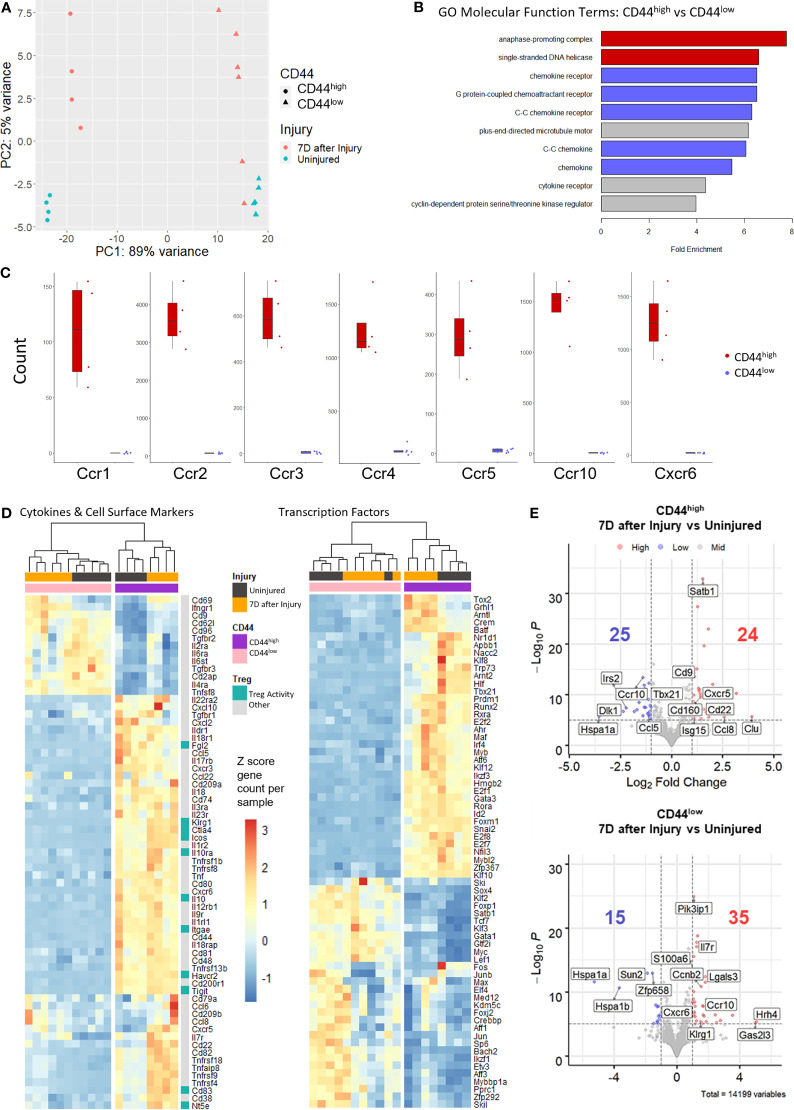
Bulk RNA sequencing of sorted CD44^high^ and CD44^low^ Tregs from the lymph nodes of uninjured and injured mice. **(A)** Principal component analysis (PCA) plots demonstrating Treg subset distribution based on gene expression from uninjured and injured mice. **(B)** Bar plots of Gene Ontology (GO) molecular function analysis of the top 2000 genes upregulated in CD44^high^ versus CD44^low^ Tregs shows enrichment of cell division (red), chemokine receptor and chemokines (blue), and other (gray) terms. **(C)** Box plots illustrating the difference in chemokine receptor gene counts between CD44^high^ and CD44^low^ Tregs. **(D)** Gene expression levels of cytokines and cell surface markers and transcription factors (rows) in injured (black bars) and uninjured (orange bars) CD44^high^ (purple bars) and CD44^low^ (pink bars) Tregs (columns) from the top 2000 genes with the highest stabilized variance. **(E)** Volcano plots of injured vs. uninjured groups plotted as Log_2_-fold change of differentially expressed (DE) genes in CD44^high^ and CD44^low^ Treg subsets. Genes with a p value less than 10^-6^ are colored blue if the gene shows a decrease of more than 2-fold and red if the gene shows an increase of more than 2-fold. Key DE genes are labeled. Counts of significantly up or down regulated genes are given with respective colors.

Next-generation RNA-sequencing (RNAseq) was used as an unbiased approach to compare gene expression variation and profiles between CD44^high^ and CD44^low^ Tregs from injured and uninjured mice. RNA from flow-sorted CD44^high^ and CD44^low^ Tregs from injured and uninjured FoxP3-GFP mice were prepared for RNA sequencing (**Figure 1E**). The resulting RNAseq data underwent quality control analysis to generate intra- and inter-group sample variability plots, sample-sample correlation plots, and sample-feature hierarchical clustering plots (**Figures S2A, B**). Principal component analysis (PCA) of sequencing data demonstrates that CD44^high^ versus CD44^low^ Tregs separated along principal component 1 (PC1) and accounted for the majority (89%) of gene expression variation (**Figure 2A**). Injury accounted for 5% of the observed variance in PC2. Gene Ontology (GO) analysis by enrichment of functional terms of DE genes indicated that genes associated with cell division and chemokine receptors were enriched in CD44^high^ as compared to CD44^low^ Tregs (**Figure 2B**). Given these findings, we specifically compared gene counts of the top differentially expressed chemokine receptors between CD44^high^ and CD44^low^ Tregs, which showed distinct differences in gene expression profiles (**Figure 2C**). Heatmaps showing expression levels of the most variably expressed cytokine, cell surface markers, and transcription factor were generated from RNAseq data to illustrate some of the key markers that distinguished these Treg subsets (**Figure 2D**). CD44^high^ Tregs expressed higher levels of known Treg-related genes such as *Icos*, *Ctla4*, *Il10ra*, *Il10*, *Itgae*, *Tigit*, *Fgl2*, *Havcr2*, *Cd83*, *Nt5e*, and *Klrg1* than CD44^low^ Tregs. Moreover, some notable cytokine and chemokine receptor genes such as *Il22ra2*, *Tgfbr1*, *Ildr1*, *Il18r1*, *Il10ra*, *Il3ra*, *Il17rb*, *Il23r*, *Il1r2*, *Il12rb*, *Ccl5*, *Cxcl10 Cxcl2*, *Cxcr3* were highly expressed in CD44^high^ Tregs, while others like *Il2ra, Il6ra, Tgfbr3, Il4ra and Trnfsf8* were highly expressed in CD44^low^ Tregs. CD44^high^ Tregs from injured mice had higher expression levels of *Cd22, Cxcr5* and *Il7r*, as well as genes encoding components of TNF receptor signaling pathways, such as *Tnfrsf18*, *Tnfrsf9*, and *Tnfrsf4*, when compared to uninjured mice. Tregs from injured mice showed higher counts of *Cd69* and *Ifnr1* in both CD44^high^ and CD44^low^ groups. Notable transcription factor genes highly expressed in CD44^high^ subsets were *Arnt2* (involved in responses to environmental stimuli), *Prdm1* (tissue-resident memory T cell signaling), *Atf6* (endoplasmic reticulum [ER]stress sensor), *Mybl2* (cell survival, proliferation, and differentiation); *Crem* (cAMP responsive element), T cell related genes (*Tbx21*, *Gata3*, *Rora*, *Irf4*, *Maf*, *Id2*, *Nfil3*), as well as cell cycle-related genes and some zinc finger proteins (**Figure 2C**). There were 24 differentially expressed (DE) genes increased and 25 DE genes decreased due to injury in the CD44^high^ subset, and 35 increased and 15 decreased in the CD44^low^ subset (**Figure 2D**). DE genes in CD44^high^ versus CD44^low^ Tregs in both the uninjured and injured groups are shown in **Figures S2C, D**. Among genes with a P value < 10^-6^, there were 1006 genes with an increase of 2-fold or more and 486 genes with a decrease of 2-fold or more in the uninjured group CD44^high^ Tregs. In the injured group, gene counts were 743 and 313, respectively. Upon burn injury, 24 genes were upregulated and 25 downregulated in the CD44^high^ Tregs, for the CD44^low^ Tregs, 35 upregulated and 15 downregulated (**Figure 2E**). Taken together, the bulk RNA sequencing results indicate that CD44^low^ and CD44^high^ Tregs are transcriptionally distinct populations.

### Injury Induces Greater Changes in T Cell Receptor (TCR)α and TCRβ Clonality and Diversity in CD44^high^ Treg Subsets

**Figure 3:**
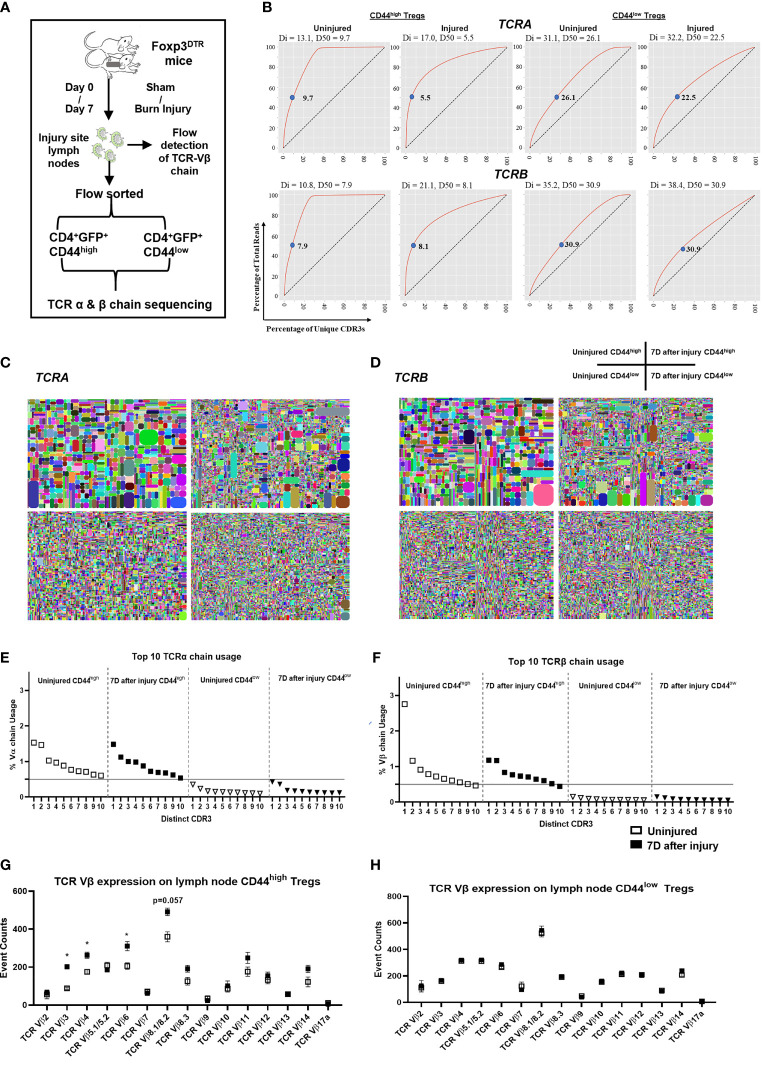
Injury induces greater changes in αβ TCR oligoclonality as well as diversity in CD44^high^ Tregs than in CD44^low^ Tregs. **(A)** Experimental scheme of TCR repertoire analysis in injured and uninjured FoxP3^DTR^ mice. **(B)** Diversity curves illustrating the diversity of TCRA and TCRB sequences in CD44^high^ and CD44^low^ Treg populations from uninjured and injured mice. Curves show the cumulative percentage of total CDR3 sequence reads as a function of the percentage of unique CDR3 sequences. Curves further from the dashed line diagonal have higher clonality. D50 values (blue dots) indicate the percentage of unique CDR3 sequences that account for 50% of the total CDR3 sequence reads. Di values are shown above each graph. Treemap plots of TCRα **(C)** and TCRβ **(D)** sequencing analysis to illustrate TCR clonotype events from TCR RNA sequencing analysis. Each TCR clonotype is represented by a colored shape and the size of the shape reflects the frequency of each CDR3 clonotype variant. Smaller shapes and more varied colors equate to greater diversity in TCR clonality. Top 10 TCR-Vα usage **(E)** and TCR-Vβ usage **(F)** among CD44^high^ and CD44^low^ Tregs. Flow cytometry analysis of TCR-Vβ chain expression on **(G)** CD44^high^ and **(H)** CD44^low^ Tregs using 14 TCR-Vβ specific antibodies. Bars represent the mean ± SEM. Data were analyzed non-parametric by multiple Mann-Whitney test and are denoted by *P<0.05 or P value compared to injured control. Data represents 3 independent experiments (n=4 mice per group).

The experimental scheme for TCRα and TCRβ repertoire analysis is shown in **Figure 3A**. Treg TCR clonality and diversity were identified by RNA sequencing using the iRepertoire analytical platform. A summary of the TCR sequencing dataset is provided in **Supplementary Table 2**. Without injury, CD44^high^ Tregs were found to have higher TCRα/TCRβ clonality than CD44^low^ Tregs as determined lower D50 and diversity index (Di) values (**Figure 3B**). The D50 value indicates the percentage of TCR CDR3 sequences that account for 50% of the total unique CDR3 sequence reads, so low D50 values would indicate high clonality (41, 42). Interestingly, the D50 value decreased for TCRα in CD44^high^ Tregs (uninjured = 9.7, injured = 5.5), but D50 for TCRβ did not change following injury suggesting that TCRα clonality is influence by injury. The Di values and curves support that CD44^high^ Tregs have lower TCR diversity than CD44^low^ Tregs (**Figure 3B**). The Di values were much lower for CD44^high^ Tregs (TCRα/13.3, TCRβ/10.8) as compared to CD44^low^ Tregs (TCRα/31.3, TCRβ/35.2). The shape of the diversity curves shown in **Figure 3B** illustrates that injury increased TCR diversity in CD44^high^ Tregs by altering the curve to be closer to the diagonal dashed line, which identifies full TCR diversity. Accordingly, CD44^low^ Tregs showed higher diversity than CD44^high^ Tregs since the diversity curves are much closer to the diagonal line. Treemap plots illustrate that injury increased the diversity of CD44^high^ Tregs, which is likely due to emergence of new CD44^high^ Tregs or movement of CD44^high^ Tregs from other tissues into lymph nodes following injury (**Figures 3C, D**). Plots comparing the top 10 TCRA and TCRB sequence percentages between CD44^high^ and CD44^low^ Tregs further support differences in TCRα and TCRβ clonality between these Treg populations (**Figures 3E, F**).

Next, specific TCR-Vβ chain expression on Tregs prepared from 2 different inbred lines of injured mice were compared by flow cytometry. Among the 14 different TCR-Vβ chains tested, Tregs expressing TCR-Vβ 3, 4, and 6 were expanded in the CD44^high^ Treg population from injured C57BL/6 mice and TCR-Vβ 4, 6, 8.1/8.2, 8.3, and 14 were expanded CD44^high^ Treg from injured BALB/c mice. No TCR-Vβ expansion was observed on CD44^low^ Tregs or conventional CD4^+^ T cells in either inbred mouse lines (**Figures 3G, H**; **Figures S3A, B**). Thus, the oligoclonal expansion of CD44^high^ Tregs is not restricted to a single inbred mouse strain with different TCR repertoires and only CD44^high^ Tregs expanded in response to injury.

### Single-Cell TCR and RNA Sequencing Identifies Injury-Induced Expanded TCR Clonotypes With Matched Transcriptome Profiles

**Figure 4:**
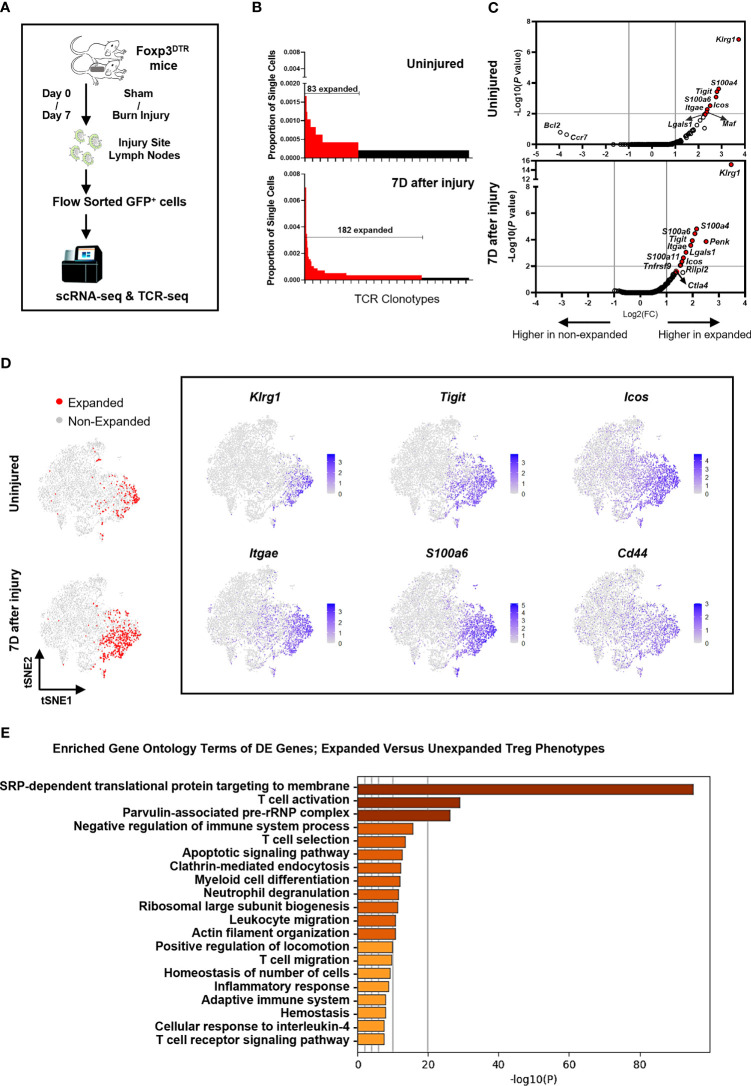
Injury induces a KLRG1^+^ Treg subset with expanded TCR clonotypes. **(A)** Experimental scheme of scRNAseq and scTCRseq analysis of FACS sorted FoxP3-GFP^+^ Tregs from the lymph nodes of mice at 7 days after sham or burn trauma injury. **(B)** Comparison of the frequencies of the top 255 TCR clonotypes in Tregs from uninjured and injured mice. Red bars depict expanded (>2) TCR clonotypes. **(C)** Volcano plots showing genes that are differentially expressed between expanded and non-expanded Tregs from uninjured and injured mice. **(D)** tSNE plots showing the location of expanded Tregs identified by TCR sequencing, as well as the expression of 6 highly differentially expressed genes in injury expanded Tregs. **(E)** Bar plots showing enriched gene ontology (GO) terms that are significantly different between clusters of expanded versus unexpanded Tregs. These GO term plots were generated using the Metascape gene annotation and analysis resource (43).

Paired single-cell TCR and RNA sequencing on FACS sorted FoxP3-GFP^+^ cells was performed to identify αβ TCR clonotypes and matched transcriptome expression profiles in Tregs from injured and uninjured mice (**Figure 4A**). Quality control was performed on single-cell RNA sequencing data to exclude doublets and dying cells (**Figure S4**). Consistent with above TCR repertoire analysis done by the iRepertoire sequencing approach, we observed 182 expanded clonotypes (2 or more paired TCRαβ clones) in Tregs from burn injured mice and 83 clonotypes in uninjured mice (**Figure 4B**). There were no overlapping paired TCR clonotypes among the top 10 clonotypes identified in CD44^high^ or CD44^low^ Tregs from injured or uninjured mice. However, the frequency of Tregs with expanded TCR clonotypes was markedly higher in injured mice as compared to uninjured mice (**Table 1**). We next classified Treg scRNAseq clusters that have 15% or more of their cells with 2 or more paired TCR clonotypes as having an “expanded phenotype” (**Figure S4E, F**). Treg-related genes such as *Klrg1*, *S100a4*, *Tigit*, *S100a6*, *Icos* and *Itgae* were found to be significantly differentially expressed in expanded as compared to non-expanded Tregs (**Figure 4C**). Moreover, t-SNE plots showed that *Klrg1*, *S100a4*, *Tigit*, *S100a6*, *Icos* and *Itgae* expression overlapped in the areas of the t-SNE cell atlas where cells with expanded TCR clonotypes were found (**Figure 4D**). Expression *Cd44* was also found to be correlative with the expanded populations. Gene ontology (GO) analysis of the top differentially expressed genes between the expanded and non-expanded populations found the most significant GO term hits to be SRP-dependent translational protein targeting to membrane, T cell activation, negative regulation of immune system process, and T cell selection (**Figure 4E**). These results suggest that injury expanded Tregs acquire a highly activated phenotype.

**Table 1:** Top 10 TCRα/β paired clonotypes in Tregs from injured and uninjured mice.

Injured	Type	V genes	J genes	C genes	CDR3s	Frequency	Proportion
**1**	TRA	TRAV13-2	TRAJ39	TRAC	CAIDRGNAGAKLTF	40	0.698%
	TRB	TRBV5	TRBJ2-7		CASSLHWGGSYEQYF		
**2**	TRA	TRAV13-1	TRAJ17	TRAC	CALAFAGNKLTF	20	0.349%
	TRB	TRBV1	TRBJ1-5	TRBC1	CTCSAPGQGNQAPLF		
**3**	TRA	TRAV9D-1	TRAJ43	TRAC	CAVSFYNNNAPRF	17	0.297%
	TRB	TRBV19	TRBJ1-6	TRBC1	CASSIGNSPLYF		
**4**	TRA	TRAV14-3	TRAJ57	TRAC	CAAGGSAKLIF	14	0.244%
	TRB	TRBV31	TRBJ2-1		CAWNWGNYAEQFF		
	TRB	TRBV4	TRBJ1-5	TRBC1	CASSPPRDRGTAPLF		
**5**	TRA	TRAV7D-4	TRAJ6	TRAC	CAASGGNYKPTF	10	0.174%
	TRB	TRBV5	TRBJ2-5		CASSPTGGEDTQYF		
**6**	TRA	TRAV4-4-DV10	TRAJ50	TRAC	CAAEASSSFSKLVF	9	0.157%
	TRB	TRBV5	TRBJ1-1	TRBC1	CASSQDTEVFF		
**7**	TRA	TRAV6-5	TRAJ7	TRAC	CALPDYSNNRLTL	9	0.157%
	TRB	TRBV19	TRBJ2-7		CASSRDWGGYEQYF		
**8**	TRA	TRAV6-6	TRAJ34	TRAC	CALGGSSNTNKVVF	9	0.157%
	TRB	TRBV12-2	TRBJ2-7		CASGDIYEQYF		
**9**	TRA	TRAV5N-4	TRAJ17	TRAC	CAAKTNSAGNKLTF	8	0.140%
	TRB	TRBV16	TRBJ2-4		CASSLDSQNTLYF		
**10**	TRA	TRAV12D-2	TRAJ57	TRAC	CALRNQGGSAKLIF	6	0.105%
	TRB	TRBV16	TRBJ2-5		CASSFKDTQYF		
**Uninjured**	**Type**	**V genes**	**J genes**	**C genes**	**CDR3s**	**Frequency**	**Proportion**
**1**	TRA	TRAV21-DV12	TRAJ40	TRAC	CILRVADTGNYKYVF	8	0.166%
	TRB	TRBV23	TRBJ1-1	TRBC1	CSSSQPGHANTEVFF		
**2**	TRA	TRAV6N-6	TRAJ6	TRAC	CALSVSGGNYKPTF	8	0.166%
	TRB	TRBV1	TRBJ2-5		CTCSAAWGQDTQYF		
**3**	TRA	TRAV4D-3	TRAJ21	TRAC	CAAEMSNYNVLYF	6	0.124%
	TRB	TRBV26	TRBJ2-7		CASSPLGGGYEQYF		
**4**	TRA	TRAV10	TRAJ50	TRAC	CAASRGASSSFSKLVF	5	0.104%
	TRB	TRBV31	TRBJ2-4		CAWSLDWVSQNTLYF		
**5**	TRA	TRAV21-DV12	TRAJ39	TRAC	CILRNNNAGAKLTF	5	0.104%
	TRB	TRBV13-3	TRBJ2-7		CASSDDSSYEQYF		
**6**	TRA	TRAV6-5	TRAJ34	TRAC	CALSSSNTNKVVF	5	0.104%
	TRB	TRBV5	TRBJ2-5		CASSQEHWGDTQYF		
**7**	TRA	TRAV7-4	TRAJ52	TRAC	CAARSNTGANTGKLTF	5	0.104%
	TRB	TRBV1	TRBJ2-3		CTCSAVWGGIETLYF		
**8**	TRA	TRAV13-4-DV7	TRAJ37		CAASGNTGKLIF	4	0.083%
	TRB	TRBV12-2	TRBJ2-2	TRAC	CASGNWGNTGQLYF		
**9**	TRA	TRAV14-1	TRAJ12		CAASAWGGYKVVF	4	0.083%
	TRB	TRBV2	TRBJ2-7	TRAC	CASSPRDRGFEQYF		
**10**	TRA	TRAV15-2-DV6-2	TRAJ34	TRAC	CALSELNTNKVVF	4	0.083%
	TRA	TRAV9-1	TRAJ34	TRAC	CAVSGPNTNKVVF		
	TRB	TRBV19	TRBJ2-5		CASSIFGGNQDTQYF		

### CyTOF Analysis Identifies CD44^high^ and CD44^low^ Treg Clusters and Validates Differentially Expressed Genes at the Protein Level

**Figure 5:**
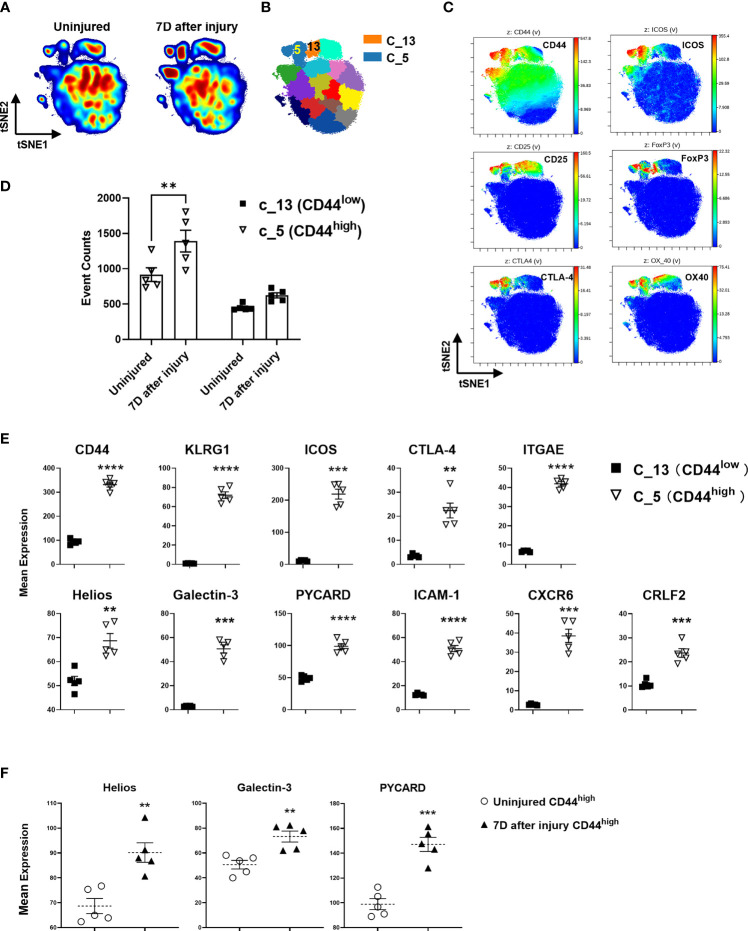
CyTOF validation of protein expression of DE genes on Treg subsets identified by RNA sequencing. **(A)** Representative density contour tSNE plots generated by 39-marker CyTOF staining of equal-sampled gated CD3^+^/CD4^+^ T cells from the lymph nodes of uninjured and injured mice, and **(B)** unsupervised computational clustering by SPADE showing phenotypic clusters as patchwork colors. Clusters 5 and 13 (C_5, C_13) are Treg subset clusters. **(C)** tSNE plots colored by the indicated antibody staining channel confirming the identification of CD44^high^ and CD44^low^ Treg subsets as well as other canonical Treg identifying markers. **(D)** Event counts from cluster 5 and 13 comparing changes in uninjured and injured mice at day 7. Bars represent the means ± SEM. Data were analyzed by two-way ANOVA, interaction P=not significant. Significant comparison by Sidak’s multiple comparisons test is denoted by ** P=0.005 (CD44^high^ cluster 5, injured versus uninjured) **(E)** Mean expression levels of select protein markers detected by CyTOF corresponding to DE genes in CD44^high^ and CD44^low^ Tregs that were identified by RNA sequencing. **(F)** The impact of injury on the expression of Helios, Galectin-3, and PYCARD in CD44^high^ Tregs 7 days after injury as measured by CyTOF staining. Bars represent the mean ± SEM of mean expression intensity levels. Data were analyzed by two-tailed unpaired t test and significance compared with uninjured control denoted by **P < 0.01, ***P < 0.001, and ****P < 0.0001. Data represent 2 independent experiments (n=5 mice per group).

Density contour plots of CyTOF staining data analyzed by tSNE for dimensionality reduction shows injury-induced increases in cell density in regions identified as FoxP3^+^ and Helios^+^ CD4^+^ T cells (**Figures 5A, C**). CyTOF data was clustered using the SPADE algorithm and cluster 5 was identified as CD44^high^ Treg and cluster 13 as CD44^low^ Treg clusters based on CD44, ICOS, CD25, Foxp3, CLTA-4 and OX40 expression patterns (**Figure 5B** and **Figures S5A, B**). The abundance of cells within the CD44^high^ Treg cluster increased at 7 days after injury but there was no significant increase in the CD44^low^ Treg cluster (**Figure 5D**). Expression of KLRG1, ICOS, CTLA-4, ITGAE (CD103), Helios, Galectin-3, PYCARD, ICAM-1, CXCR6, and CRLF2 proteins in the CD44^high^ Treg cluster was significantly higher than that in the CD44^low^ Treg cluster (**Figure 5E**). Deeper analysis of marker expression levels on Tregs showed that injury further upregulated Helios, Galectin-3 and PYCARD expression in the CD44^high^ Treg cluster (**Figure 5F**).

## Discussion

Regulatory T cells control immune tolerance and have potent counter-inflammatory functions in infectious and non-infectious diseases (9, 10, 12, 15). A better understanding of the specific functions of Tregs in reacting to and resolving tissue injuries caused by trauma, infections, tumors, and autoimmune responses will help towards designing ways to modulate Tregs as a therapeutic to resolve or enhance immune functions in these diseases. Immune reactions against non-infectious injuries are unique because they are initiated in part by the release of alarmins and DAMPs from sterile tissue and cell damage (1, 7, 9, 12). Recent findings indicate that a subpopulation of CD44^high^ Tregs react to burn trauma in mice by expanding and upregulating CTLA-4, ICOS, and GITR (17). Furthermore, this CD44^high^ Treg population demonstrated rapid activation in the lymph nodes and displayed memory-like behavior in response to injury in adoptive transfer experiments. Given the memory-like nature of CD44^high^ Tregs, we hypothesized that these Tregs might specifically react to DAMPs or other antigens that are released or exposed to the cellular immune surveillance system following tissue injury. In this report, we used advances in systems immunology technologies to perform unbiased and precise transcriptional profiling of injury reactive Tregs.

We took advantage of the FoxP3^DTR^ mouse model because all Tregs in these mice express GFP, making it possible to purify CD44^high^ and CD44^low^ Treg subpopulations for the cellular and molecular analyses performed in this project (44). In FoxP3^DTR^ mice, we validated that CD44^high^ Tregs expanded in response to injury. The CD44^low^ Tregs showed no significant increase in percentages or numbers but did show limited injury enhanced Treg mediated suppressive activity. By directly comparing the suppressive activity between CD44^high^ and CD44^low^ Tregs from injured and uninjured mice, we discovered that the CD44^high^ Treg population was much more potent at suppressing T cell proliferation than CD44^low^ Tregs; however, injury did not further enhance their suppressor potency. We suspect that this is because CD44^high^ Tregs have naturally high suppressive activity that cannot be further increased. Their specific expansion in response to injury combined with their high suppressive activity suggests that they may control autoimmune reactivity following tissue injuries.

Originally, we predicted that CD44^high^ Tregs could be protective in trauma by restraining excessive inflammation from both tissue injury and from secondary infections and sepsis (17). We decided to test this hypothesis using a clinically relevant two-hit trauma-bacterial infection mouse model (45, 46). In opposition to this hypothesis, we observed that injured Treg-depleted mice that were given CD44^high^ Tregs by adoptive transfer had higher mortality than mice that were given CD44^low^ Tregs. This outcome suggests that the persistence of highly suppressive CD44^high^ Tregs may increase susceptibility to opportunistic infections. Consistent with this, it has been demonstrated that pediatric burn patients have increased levels of circulating memory Tregs that resemble these injury responsive CD44^high^ Tregs in mice. These burn victims also showed long-term lower vaccine responses than uninjured controls, supporting our findings that injury-responsive memory Tregs are highly immunosuppressive (47). Consistent with our findings in mice, trauma patients have increased circulating levels of Tregs that can more potently suppressive T cell proliferation and patients that develop infections or sepsis showed higher circulating levels of Tregs (9, 48).

RNA sequencing of sorted CD44^high^ and CD44^low^ Tregs from injured and uninjured mice was used to discover differentially expressed (DE) genes in Treg populations, as well as those that are significantly altered by injury. We found that the 89% of the variance in gene expression was attributed to the CD44^high^ versus CD44^low^ Treg phenotype. This finding supports that CD44^high^ and CD44^low^ Tregs are transcriptionally distinct Treg subpopulations. Unsupervised hierarchical clustering of a subset of cytokine, cell surface marker, and transcription factor gene expression separated CD44^high^ and CD44^low^ Tregs into fully delineated clusters. The high expression of chemokines and chemokine receptors on CD44^high^ Tregs indicates that they are more mobile Treg population than CD44^low^ Tregs. In addition, the high expression of IL-1, IL-18, IL-12, IL-17 and TGFβ receptors strongly support that CD44^high^ Tregs are reacting to the pro-inflammatory tissue environment caused by injury. The transcription factor genes that are more highly expressed in CD44^high^ Tregs are known to control responses to environmental stimuli, ER stress sensor, cell cycle, cAMP responsive element, immune related signaling, and tissue-resident memory T cell signaling. This transcription factor expression profile is consistent with the physiological changes caused by injuries and the enriched expression of IRF4 and Blimp1 transcription factors by CD44^high^ Tregs is consistent with a prior study that elegantly showed that these transcription factors are required for Treg differentiation and suppressive function (49). Finally, CD44^high^ Tregs express higher levels of known Treg suppressive genes than CD44^low^ Tregs, consistent with the more potent suppressive activity of CD44^high^ compared to CD44^low^ Tregs. The injury modulated genes as well as those that are differentially expressed by CD44^high^ and CD44^low^ Tregs provide unique gene expression data sets for future data mining by us, and others interested in Treg biology or in modulating Tregs for immunotherapy.

Several possible mechanisms for injury-induced Treg expansion include antigen-specific T cell proliferation, pattern recognition receptor signaling by DAMPs, or response to cytokines and alarmins (50–52). In this study, we performed bulk and single-cell TCR repertoire analysis of Tregs using the iRepertoire PCR approach and the 10X Genomics platform with paired TCRα and TCRβ sequencing, respectively. Both approaches provided data to support CD44^high^ Treg activation by injury and clonal expansion. The iRepertoire TCR sequencing data indicate that TCRα and TCRβ chains are less diverse on CD44^high^ Tregs than CD44^low^ Tregs. However, 7 days after injury, the CD44^high^ Treg population showed an increase in TCRα/β chain diversity that was visualized in the Treemap plots. This finding supports that CD44^high^ Tregs react to and expand after burn trauma or traffic from tissues to immune compartments. There are two major subsets of Tregs in mice – thymus educated natural Tregs (nTreg) and induced Tregs (iTreg) that develop in peripheral tissues. We suspect that CD44^high^ Tregs are a population of nTreg with TCR bias towards recognizing self-antigens because they display low TCR diversity. Future studies will focus on delineating whether injury reactive Tregs are natural or induced Treg populations by lineage tracing or deeper molecular profiling approaches.

The exclusive expression of multiple chemokine receptors on CD44^high^ versus CD44^low^ Tregs supports the idea that CD44^high^ Tregs are trafficking Tregs with the capacity to respond to chemokines that are produced at sites of injury or inflammation. Chemokines are produced by Tregs and CCL2, CCL3, and CCL4 have been shown to be central to the trafficking of Tregs to sites of inflammation and tissues (53–55). Moreover, surgical trauma has been shown to induce CCL18 levels, which in turn increases tumor-site Tregs to further suppress tumor immunity (56). Plots comparing chemokine receptor gene expression on CD44^high^ versus CD44^low^ Tregs illustrate the exclusive expression of 7 different chemokine receptors on CD44^high^ Tregs and CyTOF staining showed that CXCR6 expression was restricted to CD44^high^ Tregs. We believe that the differential expression of chemokines and chemokine receptors by CD44^high^ Tregs plays a central role in their response to injury and strong suppressive activity.

Using bulk RNA sequencing, we were able to detect gene expression signatures in injury expanded versus unexpanded Tregs. Importantly, the genes that were significantly induced in expanded Tregs overlap with those found by bulk RNA sequencing of flow cytometry sorted CD44^high^ Tregs from injured mice. Similar sets of genes have been identified to be upregulated in Tregs in other types of injuries and diseases that induce sterile tissue injuries (9, 10, 12). In a muscular dystrophy model, clonally expanded CD44^high^ Tregs within injured skeletal muscle express higher levels of *Klrg1, Il1rl1, Itgae, Ccr2 and Ccr4* (57). Similarly, in an acute muscle injury model, *Klrg1* expressing Tregs showed TCR clonal expansion, which resulted in enhanced healing of injured muscle (58). The Tregs identified in these studies are likely similar to those identified in our mouse burn trauma model and appear to be beneficial in injury resolution. We suspect that CD44^high^ Tregs in our burn trauma model play a similar role in resolving injury-induced tissue damage to promote healing.

We identified *Klrg1* gene expression in injury expanded Treg clonotypes and validated KLRG1 protein expression on CD44^high^, but not CD44^low^ Tregs. KLRG1^+^ Tregs have been identified in the tumor microenvironment of murine models and human samples of non-small cell lung cancer (59–61). Consistent with our findings, these KLRG1^+^ Tregs were strongly immunosuppressive (60). Severe COVID-19 patients that develop lung injury showed expanded KLRG1^+^ Tregs that express *Cxcr3*, *Il10*, *Il12b1*, *Ilrb1*, *Tbx21*, *Gata3* gene signatures similar to what was found in this study (62). Though KLRG1^+^ Tregs are normally potent immune suppressive cells, they have also been identified as being dysfunctional in autoimmune mouse models. For example, in the non-obese diabetic (NOD) mouse model, KLRG1^+^ Tregs were not suppressive and tended to differentiate into Th1-or Th17-like effector cells in the pancreas causing heightened disease (63). Similarly, in a TCR transgenic NOD mouse model, KLRG1^+^ Tregs were unable to suppress the development of type 1 diabetes (64). Thus, KLRG1^+^ Tregs represent a Treg population with plasticity that contributes to the pathogenesis of infections, cancer, and autoimmune diseases. We speculate that tissue damage might be a common stimulus for Treg activation and differentiation in these sites of infection or injury.

In summary, we identify CD44^high^ Tregs as a potently immunosuppressive population that expand in an oligoclonal manner in response to injury. Molecular profiling of CD44^high^ versus CD44^low^ Tregs indicates that they are distinct subsets with different transcriptional and functional profiles. We have not yet identified antigens or factors that contribute to their expansion. However, future studies will use the paired TCRαβ sequence information gained from injury expanded Treg clonotypes to identify antigens and antigen presenting cells that activate CD44^high^ Tregs. We propose that the activation and expansion of injury responsive Tregs is a fundamental feature of many pathological immune responses that cause tissue damage. Thus, we anticipate that these data will contribute valuable information that will help with designing novel Treg targeted therapies to modulate their behavior in trauma, infections, cancer, and autoimmunity.

## Data Availability Statement

The datasets presented in this study can be found in online repositories. The names of the repository/repositories and accession number(s) can be found below: https://www.ncbi.nlm.nih.gov/geo/, accession ID: GSE174790.

## Ethics Statement

The animal study was reviewed and approved by Brigham and Women’s Hospital IACUC.

## Author Contributions

Conceptualization: JL and FG; Methodology: FG, HL, KH, JK, AC, and FZ; Investigation: FG and HL; Visualization: FG, BH, and AG; Funding acquisition: JL; Project administration: JL and JK; Supervision: JL; Writing – original draft: FG; Writing, review, editing: AG, LC, JN, and JL. All authors contributed to the article and approved the submitted version.

## Funding

National Institutes of Health grant AI092905 and AI148232 (JL).

## Conflict of Interest

The authors declare that the research was conducted in the absence of any commercial or financial relationships that could be construed as a potential conflict of interest.

## Publisher’s Note

All claims expressed in this article are solely those of the authors and do not necessarily represent those of their affiliated organizations, or those of the publisher, the editors and the reviewers. Any product that may be evaluated in this article, or claim that may be made by its manufacturer, is not guaranteed or endorsed by the publisher.

## Supplementary Material

Supplementary Figure 1 CD44^high^ Tregs decrease survival in a Treg-depleted mouse model of trauma injury with secondary lung infection. **(A)** Experimental scheme. C57BL/6 mice were subjected to 20% total body surface area burn injury. Foxp3^DTR^ mice were treated with 40ng/kg diphtheria toxin (DT) at 2h and 24h after burn injury to deplete Tregs. 7 days after injury, CD44^high^ or CD44^low^ CD4^+^CD25^+^ cells (Tregs) were FACS sorted from injury-site draining lymph nodes (axillary, brachial, and inguinal). Tregs were transfused into the injured Foxp3^DTR^ mice at 50,000 cells/mouse by intracardiac injection. The mice were subsequently challenged with intranasal *Pseudomonas aeruginosa* 1 day after Treg-transfusion. Survival was monitored over a week period. **(B)** Injury-site draining lymph nodes, spleen, and blood were collected from Treg depleted Foxp3^DTR^ mice 24 hours after last injection to determine Treg depletion efficiency by flow cytometry. **(C)** Survival of mice [n=14 (CD44^high^), n=24 (CD44^low^)] subjected to burn injury with secondary *P. aeruginosa* pulmonary infection. Data are presented as a Kaplan-Meier survival curve and analyzed by Log-rank (Matel-Cox) test

Supplementary Figure 2 Bulk RNA sequencing of FACS sorted CD44^high^ and CD44^low^ Tregs in injury-site lymph nodes of mice subjected to burn/sham injury. **(A)** Sample-Sample Clustering Map demonstrating the correlation values between samples. Groups are annotated at the top. **(B)** Sample-Feature (Gene) Hierarchical Clustering Map with samples along the x-axis and genes along the y-axis. Metadata columns are annotated along the top. Volcano plots showing the log_2_ fold change of differentially expressed genes in CD44^high^ and CD44^low^ Treg subsets in **(C)** uninjured and **(D)** injured mice. Blue dots represent the genes in CD44^low^ Tregs with a more than 2-fold increase compared to CD44^high^ Tregs. Red dots indicate genes with a more than 2-fold increase in CD44^high^ Tregs compared to CD44^low^ Tregs [n=4 (uninjured CD44^high^ Tregs, 7D after injury CD44^high^ Tregs and uninjured CD44^low^ Tregs), n=6 (7D after injury CD44^low^ Tregs)].

Supplementary Figure 3 TCRvβ clonotype analysis demonstrating expansion only in CD44^high^ Tregs. TCRvβ staining shows that the clonotype expansion occurs on CD44^high^ Tregs but not on other cell types, in both **(A)** C57BL/6 (n=4 injured or uninjured mice) and **(B)** BALB/c mice (n=5 injured or uninjured mice). Bars represent the means ± SEM. Data were analyzed non-parametric by multiple Mann-Whitney test and were denoted by * P<0.05 or ** P<0.01 compared to injured control. Data represents 3 independent experiments.

Supplementary Figure 4 Single Cell Quality Metrics. **(A)** Sequencing saturation plot showing the Sequencing Saturation metric as a function of down-sampled sequencing depth (measured in mean reads per cell), up to the observed sequencing depth. Sequencing Saturation is a measure of the observed library complexity and approaches 1.0 (100%) when all converted mRNA transcripts have been sequenced. The slope of the curve near the endpoint can be interpreted as an upper bound to the benefit to be gained from increasing the sequencing depth beyond this point. The dotted line is drawn at a value reasonably approximating the saturation point. **(B)** Median genes per cell shows the median genes per cell as a function of down-sampled sequencing depth in mean reads per cell, up to the observed sequencing depth. The slope of the curve near the endpoint can be interpreted as an upper bound to the benefit to be gained from increasing the sequencing depth beyond this point. Violin plots demonstrating **(C)** number of unique features (genes) and **(D)** percentage of reads mapped to the mitochondrial genome. Dotted red lines show the cutoffs used for final data analysis. Cells with greater than 3000 or less than 200 genes, or cells with greater than 6% mitochondrial reads were filtered out. **(E)** tSNE plot showing the Seurat clusters used to group the expanded and unexpanded phenotypes. **(F)** tSNE showing the cells grouped into expanded and unexpanded phenotypes as defined by clusters containing at least 15% of single cells with >2 identical paired CDR3 sequences.

Supplementary Figure 5 Identification of clusters identified by CyTOF stains from equal-sampled CD3^+^CD4^+^ T cells. **(A)** A heatmap of calculated ArcSinh ratio of mean expression levels of markers in each cluster controlled by row’s minimum. **(B)** tSNE plots colored by channel showing the expression of the markers. Data are from equal-sampled CD3^+^CD4^+^ T cells of concatenated files. **(C)** Volcano plot showing translated proteins of differentially expressed genes between CD44^high^ and CD44^low^ Tregs.

Supplementary Figure 6 Flow cytometry gating schemes. Representative flow cytometry plots demonstrating gating for CD4^+^ and CD4^-^ T cells, CD44^high^ and CD44^low^ Tregs, and TCRVβ^+^ populations.

Supplementary Figure 7 FACS sort plots. Representative FACS sort plots of **(A)** CD4^+^CD44^high^GFP^+^ (CD44^high^ Tregs) and CD4^+^CD44^low^GFP^+^ (CD44^low^Tregs) for bulk RNAseq and iRepertoire analysis of TCRα and TCRβ, and **(B)** Sorting scheme used to prepare GFP^+^(Tregs) for single cell mRNA sequencing analysis.

Supplementary Table 1 Details of RNA-based TCR repertoire sequencing samples used for iRepertoire analysis.

Supplementary Table 2 Sample information of RNA-based TCR repertoire sequencing.

Supplementary Table 3 Sample statistics of scRNAseq analysis.

Supplementary Table 4 CyTOF antibodies panel.
