# Supplementary figures and images for: Distinct Injury Responsive Regulatory T Cells Identified by Multi-Dimensional Phenotyping
Source: Front Immunol. 2022 May 12;13:833100. doi: 10.3389/fimmu.2022.833100 (PMC9135044; doi:10.3389/fimmu.2022.833100)

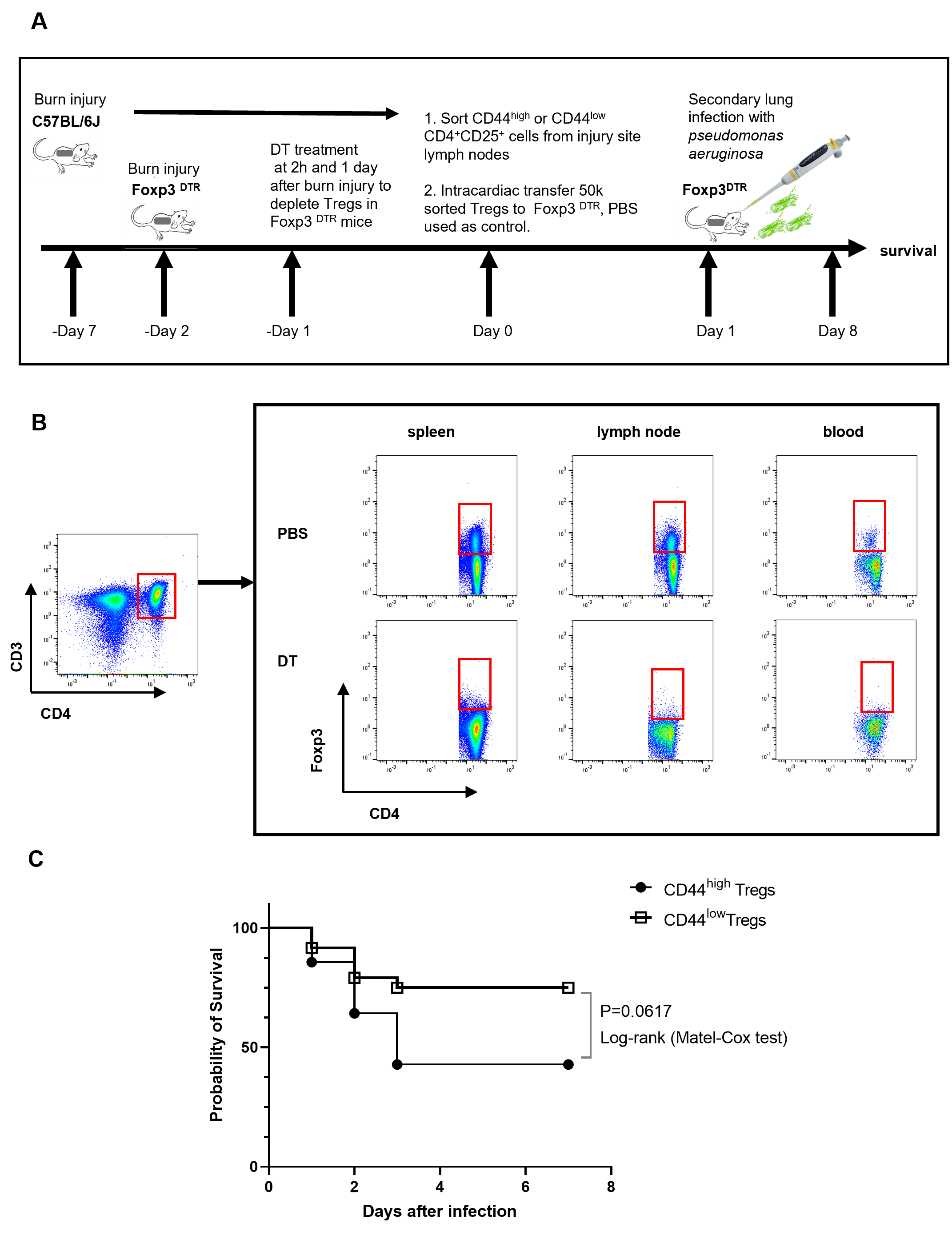

Supplement: Supplementary Figure 1 — CD44high Tregs decrease survival in a Treg-depleted mouse model of trauma injury with secondary lung infection. (A) Experimental scheme. C57BL/6 mice were subjected to 20% total body surface area burn injury. Foxp3DTR mice were treated with 40ng/kg diphtheria toxin (DT) at 2h and 24h after burn injury to deplete Tregs. 7 days after injury, CD44high or CD44low CD4+CD25+ cells (Tregs) were FACS sorted from injury-site draining lymph nodes (axillary, brachial, and inguinal). Tregs were transfused into the injured Foxp3DTR mice at 50,000 cells/mouse by intracardiac injection. The mice were subsequently challenged with intranasal Pseudomonas aeruginosa 1 day after Treg-transfusion. Survival was monitored over a week period. (B) Injury-site draining lymph nodes, spleen, and blood were collected from Treg depleted Foxp3DTR mice 24 hours after last injection to determine Treg depletion efficiency by flow cytometry. (C) Survival of mice [n=14 (CD44high), n=24 (CD44low)] subjected to burn injury with secondary P. aeruginosa pulmonary infection. Data are presented as a Kaplan-Meier survival curve and analyzed by Log-rank (Matel-Cox) test [file Image_1.tif]

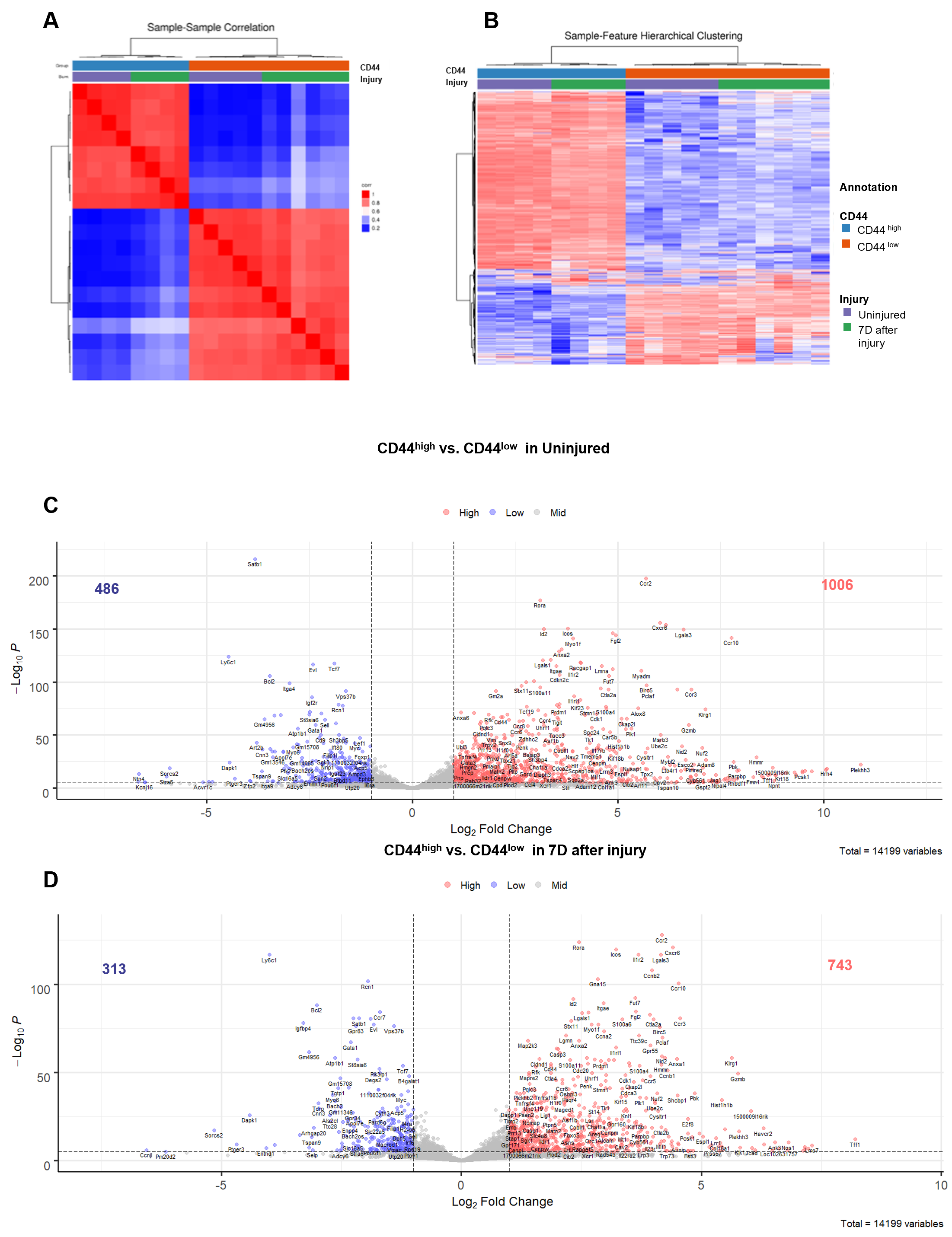

Supplement: Supplementary Figure 2 — Bulk RNA sequencing of FACS sorted CD44high and CD44low Tregs in injury-site lymph nodes of mice subjected to burn/sham injury. (A) Sample-Sample Clustering Map demonstrating the correlation values between samples. Groups are annotated at the top. (B) Sample-Feature (Gene) Hierarchical Clustering Map with samples along the x-axis and genes along the y-axis. Metadata columns are annotated along the top. Volcano plots showing the log2 fold change of differentially expressed genes in CD44high and CD44low Treg subsets in (C) uninjured and (D) injured mice. Blue dots represent the genes in CD44low Tregs with a more than 2-fold increase compared to CD44high Tregs. Red dots indicate genes with a more than 2-fold increase in CD44high Tregs compared to CD44low Tregs [n=4 (uninjured CD44high Tregs, 7D after injury CD44high Tregs and uninjured CD44low Tregs), n=6 (7D after injury CD44low Tregs)]. [file Image_2.tif]

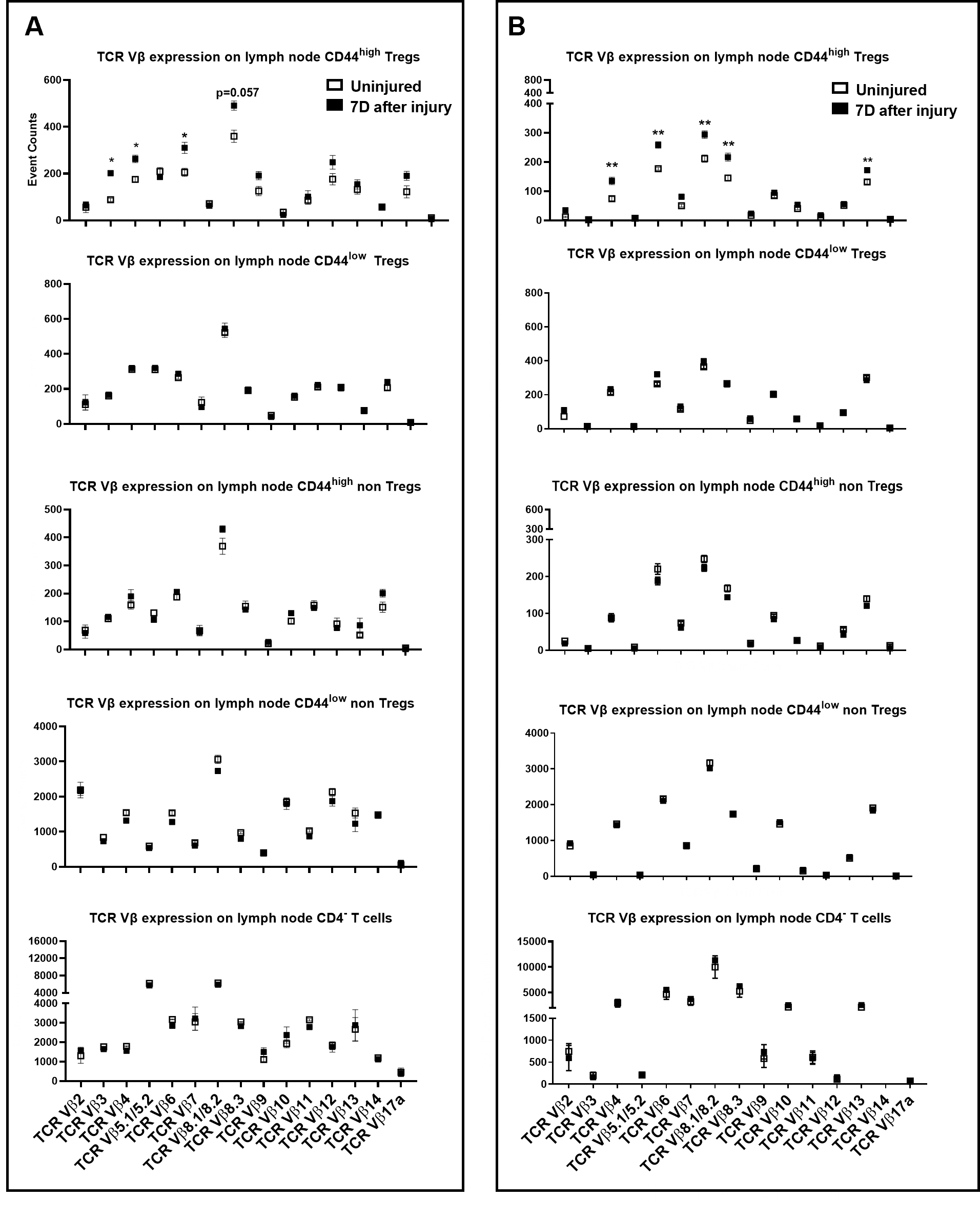

Supplement: Supplementary Figure 3 — TCRvβ clonotype analysis demonstrating expansion only in CD44high Tregs. TCRvβ staining shows that the clonotype expansion occurs on CD44high Tregs but not on other cell types, in both (A) C57BL/6 (n=4 injured or uninjured mice) and (B) BALB/c mice (n=5 injured or uninjured mice). Bars represent the means ± SEM. Data were analyzed non-parametric by multiple Mann-Whitney test and were denoted by * P<0.05 or ** P<0.01 compared to injured control. Data represents 3 independent experiments. [file Image_3.tif]

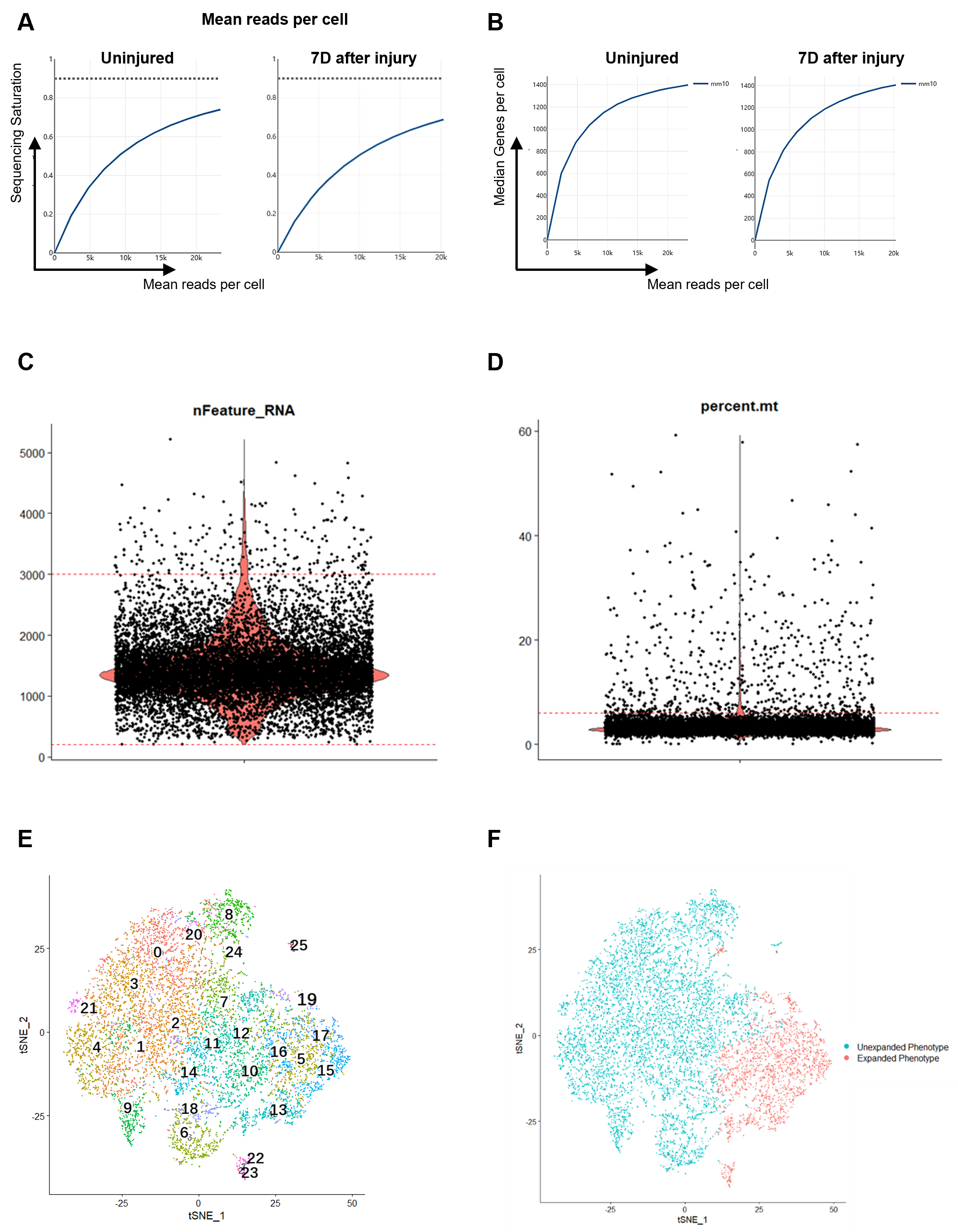

Supplement: Supplementary Figure 4 — Single Cell Quality Metrics. (A) Sequencing saturation plot showing the Sequencing Saturation metric as a function of down-sampled sequencing depth (measured in mean reads per cell), up to the observed sequencing depth. Sequencing Saturation is a measure of the observed library complexity and approaches 1.0 (100%) when all converted mRNA transcripts have been sequenced. The slope of the curve near the endpoint can be interpreted as an upper bound to the benefit to be gained from increasing the sequencing depth beyond this point. The dotted line is drawn at a value reasonably approximating the saturation point. (B) Median genes per cell shows the median genes per cell as a function of down-sampled sequencing depth in mean reads per cell, up to the observed sequencing depth. The slope of the curve near the endpoint can be interpreted as an upper bound to the benefit to be gained from increasing the sequencing depth beyond this point. Violin plots demonstrating (C) number of unique features (genes) and (D) percentage of reads mapped to the mitochondrial genome. Dotted red lines show the cutoffs used for final data analysis. Cells with greater than 3000 or less than 200 genes, or cells with greater than 6% mitochondrial reads were filtered out. (E) tSNE plot showing the Seurat clusters used to group the expanded and unexpanded phenotypes. (F) tSNE showing the cells grouped into expanded and unexpanded phenotypes as defined by clusters containing at least 15% of single cells with >2 identical paired CDR3 sequences. [file Image_4.tif]

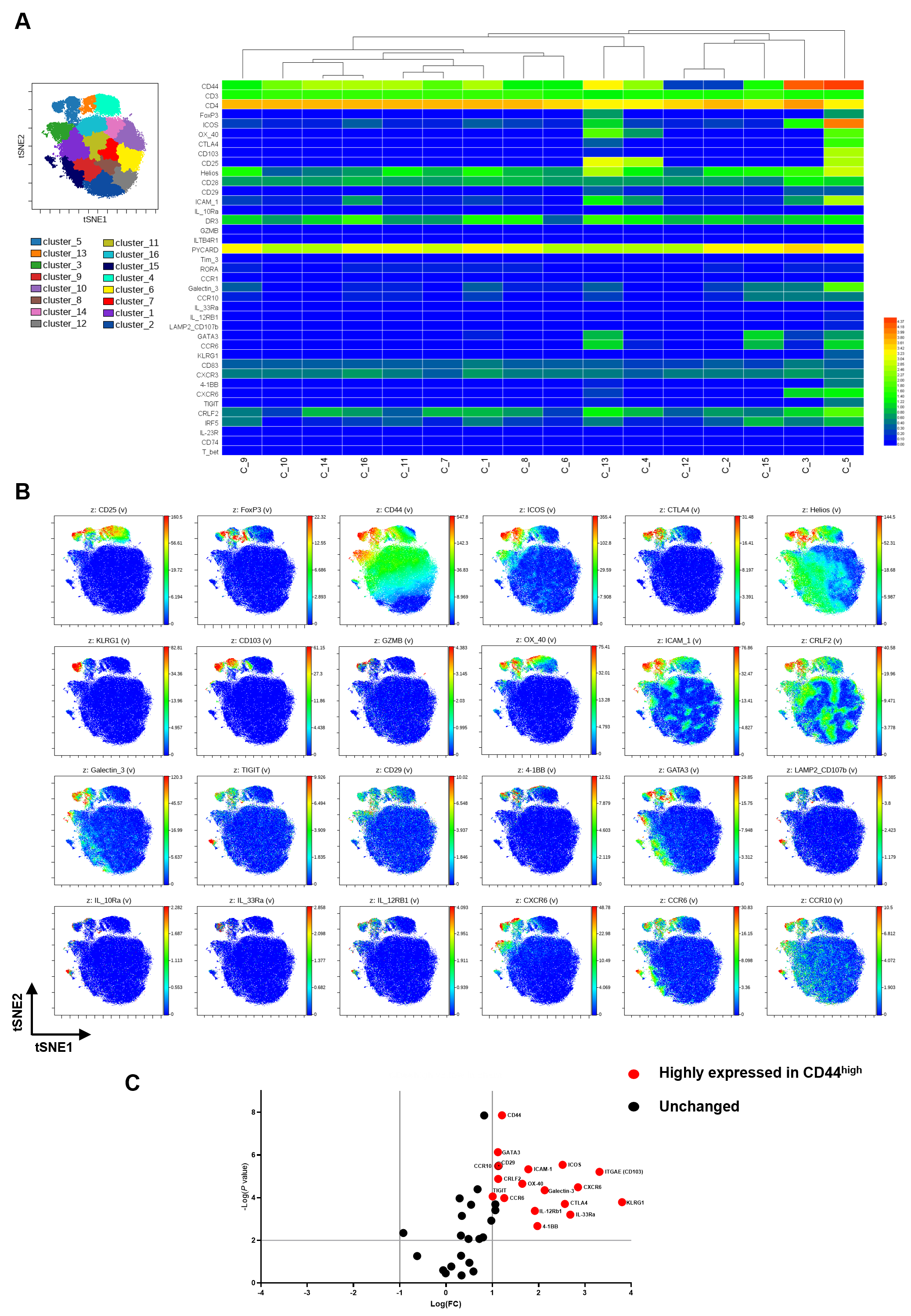

Supplement: Supplementary Figure 5 — Identification of clusters identified by CyTOF stains from equal-sampled CD3+CD4+ T cells. (A) A heatmap of calculated ArcSinh ratio of mean expression levels of markers in each cluster controlled by row’s minimum. (B) tSNE plots colored by channel showing the expression of the markers. Data are from equal-sampled CD3+CD4+ T cells of concatenated files. (C) Volcano plot showing translated proteins of differentially expressed genes between CD44high and CD44low Tregs. [file Image_5.tif]

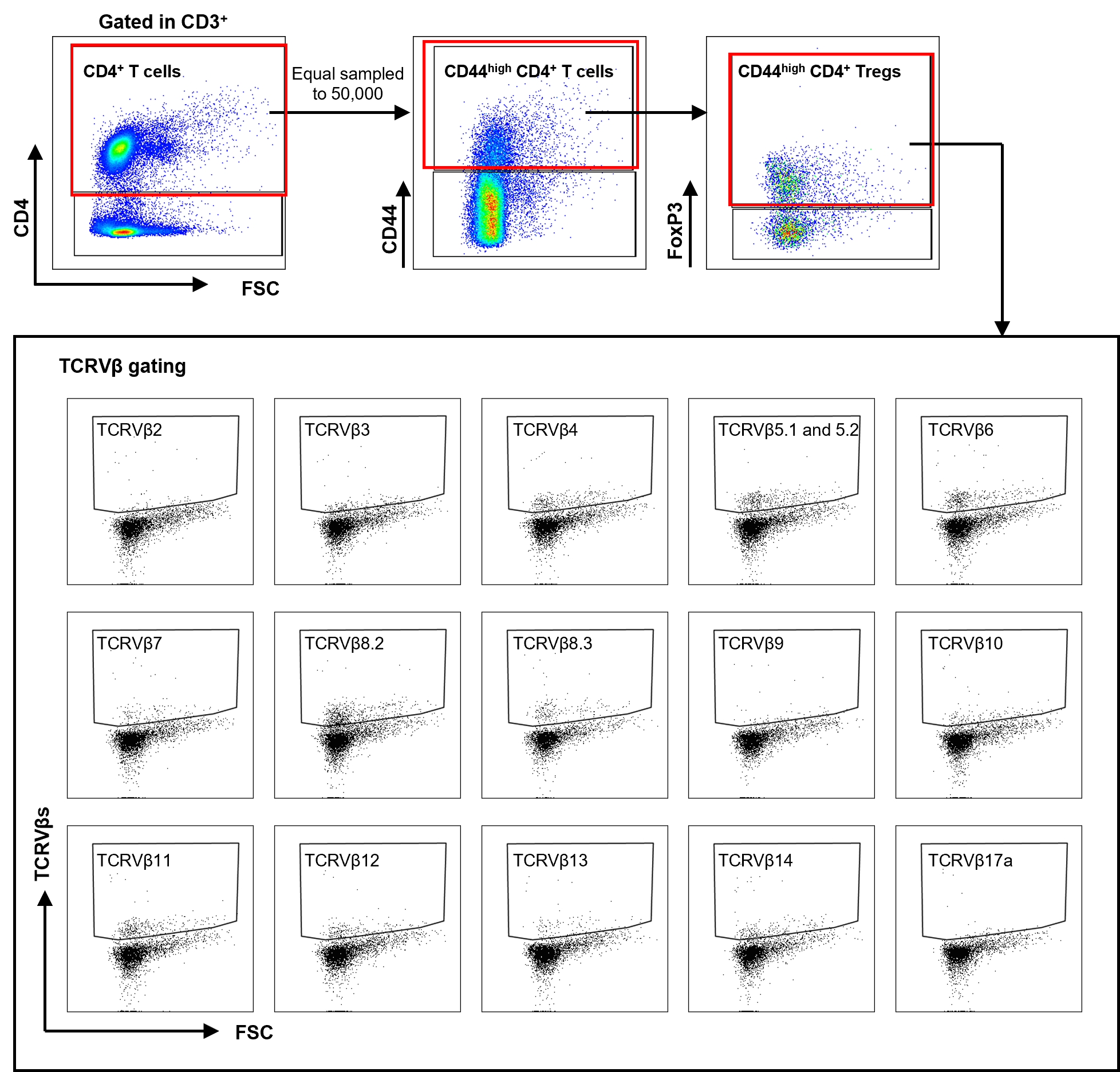

Supplement: Supplementary Figure 6 — Flow cytometry gating schemes. Representative flow cytometry plots demonstrating gating for CD4+ and CD4- T cells, CD44high and CD44low Tregs, and TCRVβ+ populations. [file Image_6.tif]

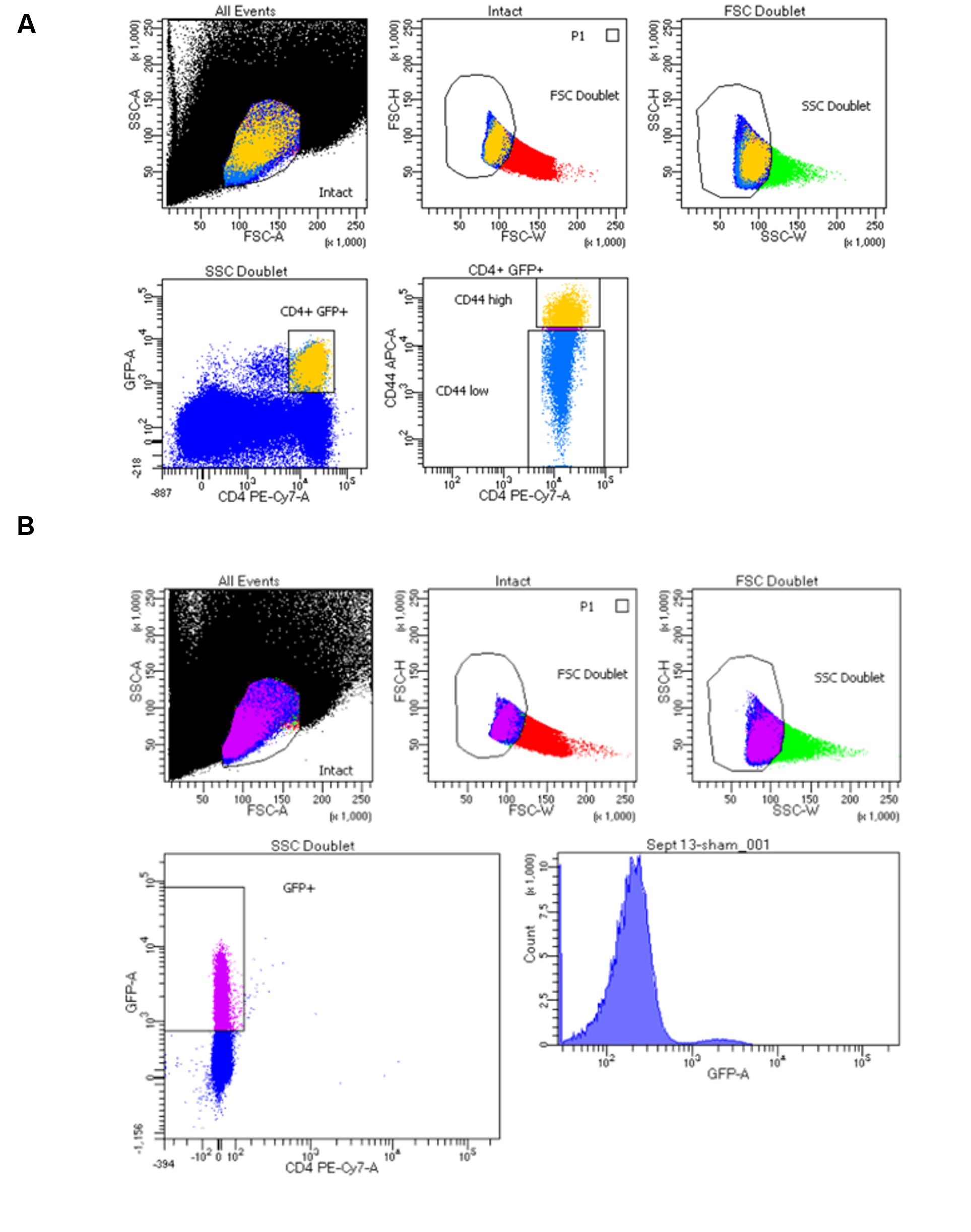

Supplement: Supplementary Figure 7 — FACS sort plots. Representative FACS sort plots of (A) CD4+CD44highGFP+ (CD44high Tregs) and CD4+CD44lowGFP+ (CD44lowTregs) for bulk RNAseq and iRepertoire analysis of TCRα and TCRβ, and (B) Sorting scheme used to prepare GFP+(Tregs) for single cell mRNA sequencing analysis. [file Image_7.tif]
