# Supplementary material for: Distinct Injury Responsive Regulatory T Cells Identified by Multi-Dimensional Phenotyping
Source: Front Immunol. 2022 May 12;13:833100. doi: 10.3389/fimmu.2022.833100 (PMC9135044; doi:10.3389/fimmu.2022.833100)
Supplement: Supplementary Table 1 — Details of RNA-based TCR repertoire sequencing samples used for iRepertoire analysis. [file Table_1.pdf]

Supplementary Table 1. Details of raw RNA-based TCR repertoire sequencing data

Sample	Sample id	Chain	Reads	CDR3	Unique CDR3	D50	Diversity Index	Entropy	Cell numbers (X10^3)	RNA extract(ng/μl)
7D after injury CD44^{high} Tregs (BH)	158770	TRA	610197	610197	13120	5.3	16.9	11.5	352	14.1
7D after injury CD44^{high} Tregs (BH) repeat	158771	TRA	543985	543985	10693	5.5	17	11.5	352	14.1
7D after injury CD44^{low} Tregs(BL)	158765	TRA	301141	301141	11086	22.5	32.2	12.8	485	10.68
7D after injury CD44^{low} Tregs(BL) repeat	158767	TRA	355085	355085	10138	21.4	30.4	12.8	485	10.68
Uninjured CD 44^{high} Tregs (SH)	158769	TRA	547155	547155	2339	9.7	13.3	9.4	73	5.64
Uninjured CD 44^{high} Tregs (SH) repeat	158772	TRA	549768	549768	2279	8.7	11.9	9.2	73	5.64
Uninjured CD 44^{low} Tregs (SL)	158768	TRA	325899	325899	4297	26.1	31.1	11.6	288	12.23
Uninjured CD 44^{low} Tregs (SL) repeat	158773	TRA	462723	462723	6024	25.2	30.8	12.1	288	12.23
7D after injury CD44^{high} Tregs (BH)	158763	TRB	587382	587382	16743	8.1	21.1	11.9	352	14.1
7D after injury CD44^{high} Tregs (BH) repeat	158762	TRB	697880	697880	15078	8.5	21.6	11.9	352	14.1
7D after injury CD44^{low} Tregs(BL)	158758	TRB	647467	647467	24036	30.9	38.4	13.1	485	10.68
7D after injury CD44^{low} Tregs(BL) repeat	158760	TRB	814686	814686	24560	31.5	38.8	13.1	485	10.68
Uninjured CD 44^{high} Tregs (SH)	158759	TRB	634112	634112	3375	7.9	10.8	9.6	73	5.64
Uninjured CD 44^{high} Tregs (SH) repeat	158764	TRB	735518	735518	3676	7.4	10	9.6	73	5.64
Uninjured CD 44^{low} Tregs (SL)	158761	TRB	894532	894532	11596	30.9	35.2	13	288	12.23
Uninjured CD 44^{low} Tregs (SL) repeat	158766	TRB	1020265	1020265	13798	35.6	40.4	13.2	288	12.23
