# Supplementary material for: Distinct Injury Responsive Regulatory T Cells Identified by Multi-Dimensional Phenotyping
Source: Front Immunol. 2022 May 12;13:833100. doi: 10.3389/fimmu.2022.833100 (PMC9135044; doi:10.3389/fimmu.2022.833100)
Supplement: Supplementary Table 2 — Sample information of RNA-based TCR repertoire sequencing. [file Table_2.pdf]

Supplementary Table 2. Sample information of RNA-based TCR repertoire sequencing

Sample names	cell events	RNA extraction (ng/ml)
7D after injury CD44^{high} Tregs (BH)	352,000	14.1
7D after injury CD44^{low} Tregs(BL)	485,000	10.7
Uninjured CD 44^{high} Tregs (SH)	73,000	5.6
Uninjured CD 44^{low} Tregs (SL)	288,000	12.2
