# Supplementary material for: Distinct Injury Responsive Regulatory T Cells Identified by Multi-Dimensional Phenotyping
Source: Front Immunol. 2022 May 12;13:833100. doi: 10.3389/fimmu.2022.833100 (PMC9135044; doi:10.3389/fimmu.2022.833100)
Supplement: Supplementary Table 3 — Sample statistics of scRNAseq analysis. [file Table_3.pdf]

Supplementary Table 3. Sample statistics of scRNA-seq analysis

Sample name	Cell Ranger version	Estimated Number of Cells	Mean Reads per Cell	Median Genes per Cell	Number of Reads	Sequencing Saturation	Total Genes Detected	Median UMI Counts per Cell
Uninjured	3.1.0	5,284	23,379	1,398	123,535,033	74.00%	15,357	3,710
7D after Injury	3.1.0	6,312	20,297	1,405	128,115,793	68.70%	15,688	3,754
