# Supplementary material for: Distinct Injury Responsive Regulatory T Cells Identified by Multi-Dimensional Phenotyping
Source: Front Immunol. 2022 May 12;13:833100. doi: 10.3389/fimmu.2022.833100 (PMC9135044; doi:10.3389/fimmu.2022.833100)
Supplement: Supplementary Table 4 — CyTOF antibodies panel. [file Table_4.pdf]

Supplementary Table 4. CyTOF antibodies panel

Ab #	Mouse Marker	Clone	Metal	Subcellular location	Vender
1	T-bet	4b10	111Cd	Intracellular	BioLegend
2	CD3	145-2C11	115In	Surface	BioLegend
3	CD44	IM7	141Pr	Surface	BioLegend
4	PYCARD(ASC/TMS1)	D2W8U	142Nd	Intracellular	CST
5	Tim3	5D12	143Nd	Surface	EMD Millipore
6	RORA	6G4C18	144Nd	Intracellular	BioLegend
7	CD4	RM4-5	145Nd	Surface	BioLegend
8	CXCR3(CD183)	220803	146Nd	Surface	R&D
9	CD28	E18	147Sm	Surface	BioLegend
10	ICAM1(CD54)	3-E2	148Nd	Surface	BD
11	CCR1	643854	149Sm	Surface	R&D
12	CD137(4-1BB)	3H3	150Nd	Surface	BioXcell
13	Granzyme B	GB11	151Eu	Intracellular	BioLegend
14	LTB4R1	Polyclonal	152Sm	Surface	ThermoFisher
15	CTLA-4	UC10-4B9	153Eu	Surface	BioLegend
16	ITGAE (CD103)	2-E7	154Sm	Surface	BioLegend
17	LGALS3 (Galectin3)	M3/38	155Gd	Intracellular	BioLegend
18	IL10RA	105653	156Gd	Surface	R&D
19	CXCR6(CD186)	SA051D1	157Gd	Surface	BioLegend
20	CCR10	248918	158Gd	Surface	R&D
21	IL-33Ra(IL1RL1/ST2)	DIH9	159Tb	Surface	BioLegend
22	IL12RB1	Polyclonal	160Gd	Surface	R&D
23	CD83	Michel-19	161Dy	Surface	BioLegend
24	FoxP3	FJK-16s	162Dy	Surface	eBioscience
25	TIGIT	1G9	163Dy	Intracellular	BioLegend
26	LAMP2(CD107b)	M3/84	164Dy	Surface	BioLegend
27	TNFRSF25 (DR3)	4C12	165Ho	Surface	BioLegend
28	GATA3	TWAJ	166Er	Intracellular	eBioscience
29	CD25(IL-2Ra)	3C7	167Er	Surface	BioLegend
30	Helios	22F6	168Er	Intracellular	BioLegend
31	CCR6	29-2L17	169Tm	Surface	BioLegend
32	CD278(ICOS)	C398.4A	170Er	Surface	BioLegend
33	CRLF2(TSLPR)	Polyclonal	171Yb	Surface	R&D
34	KLRG1	2F1/KLRG1	172Yb	Surface	BioLegend
35	IRF5	903430	173Yb	Intracellular	R&D
36	CD134(OX40,TNFRSF4)	OX-86	174Yb	Surface	BioLegend
37	CD29	HMB-1	175Lu	Surface	BioLegend
38	IL-23R	12B2B64	176Yb	Surface	BioLegend
39	CD74	In1	209Bi	Intracellular	BioLegend
